# Experimental validation of computerised models of clustering of platelet glycoprotein receptors that signal via tandem SH2 domain proteins

**DOI:** 10.1371/journal.pcbi.1010708

**Published:** 2022-11-28

**Authors:** Zahra Maqsood, Joanne C. Clark, Eleyna M. Martin, Yam Fung Hilaire Cheung, Luis A. Morán, Sean E. T. Watson, Jeremy A. Pike, Ying Di, Natalie S. Poulter, Alexandre Slater, Bodo M. H. Lange, Bernhard Nieswandt, Johannes A. Eble, Mike G. Tomlinson, Dylan M. Owen, David Stegner, Lloyd J. Bridge, Christoph Wierling, Steve P. Watson

**Affiliations:** 1 Institute of Cardiovascular Sciences, IBR Building, College of Medical and Dental Sciences, University of Birmingham, Birmingham, United Kingdom; 2 Alacris Theranostics, GmbH, Berlin, Germany; 3 Rudolf Virchow Center for Integrative and Translation Bioimaging, University of Würzburg and Institute of Experimental Biomedicine I, University Hospital of Würzburg, Würzburg, Germany; 4 Centre of Membrane Proteins and Receptors (COMPARE), Universities of Birmingham and Nottingham, Birmingham, United Kingdom; 5 Leibniz-Institut für Analytische Wissenschaften–ISAS—e. V., Dortmund, Germany; 6 School of Biochemistry, Cardiovascular Research Institute Maastricht (CARIM), Maastricht University, Maastricht, The Netherlands; 7 Institute of Physiological Chemistry and Pathobiochemistry, University of Münster, Münster, Germany; 8 Department of Biosciences, University of Birmingham, Birmingham, United Kingdom; 9 Institute of Immunology and Immunotherapy, IBR Building, College of Medical and Dental Sciences, University of Birmingham, Birmingham, United Kingdom; 10 Faculty of Environment & Technology, Department of Computer Science and Creative Technologies, University of the West England, Bristol, United Kingdom; Heidelberg Institute for Theoretical Studies (HITS gGmbH), GERMANY

## Abstract

The clustering of platelet glycoprotein receptors with cytosolic YxxL and YxxM motifs, including GPVI, CLEC-2 and PEAR1, triggers activation via phosphorylation of the conserved tyrosine residues and recruitment of the tandem SH2 (Src homology 2) domain effector proteins, Syk and PI 3-kinase. We have modelled the clustering of these receptors with monovalent, divalent and tetravalent soluble ligands and with transmembrane ligands based on the law of mass action using ordinary differential equations and agent-based modelling. The models were experimentally evaluated in platelets and transfected cell lines using monovalent and multivalent ligands, including novel nanobody-based divalent and tetravalent ligands, by fluorescence correlation spectroscopy. Ligand valency, receptor number, receptor dimerisation, receptor phosphorylation and a cytosolic tandem SH2 domain protein act in synergy to drive receptor clustering. Threshold concentrations of a CLEC-2-blocking antibody and Syk inhibitor act in synergy to block platelet aggregation. This offers a strategy for countering the effect of avidity of multivalent ligands and in limiting off-target effects.

## Author summary

All cells in the body have proteins in the membrane known as receptors which have the ability to recognise a wide variety of extracellular stimuli. This interaction generates signals into the cell which control cell behaviour. In this study, we have used two different computational methods to predict how the binding of stimuli leads to the clustering of a subset of receptors which is required to generate the signals. We have then tested the models using advanced microscopy methods and by the generation of novel stimuli. Our results show that clustering of receptors on the cell surface can be achieved through at least 3 mechanisms that work together to generate large clusters which induce powerful signals. Over 70% of drugs target receptors on the cell surface. However, many have side-effects that can limit their use in patients. We show that simultaneously targeting 2 of the mechanisms reduces the concentration of drug needed for inhibition thus reducing the side-effects.

## Introduction

Platelets are regulated by two major classes of surface receptors: seven transmembrane receptors, which signal through heterotrimeric G proteins, and glycoprotein receptors, which signal through tyrosine kinases. Current antiplatelet drugs such as aspirin and P2Y_12_ receptor antagonists target G protein-coupled receptor (GPCR) regulated pathways but are not effective in all individuals and cause a significant increase in risk of bleeding in some people and this can be life-threatening. The glycoprotein receptors GPVI, CLEC-2 and PEAR1 are considered to be targets for a new class of antithrombotic agents based on mouse models and genome-wide association studies with inhibition predicted to cause less bleeding than current antiplatelet agents [1,2]. These receptors are activated by clustering, and antagonists must therefore overcome the net effect of ligand affinity and avidity for effective inhibition, thus favouring inhibitors with high affinity and a slow off-rate of dissociation. A deeper understanding of the relationship between ligand engagement and receptor clustering of this class of receptor will guide development of more potent antithrombotic agents.

The major signalling glycoprotein receptors in platelets have tyrosine-based signalling motifs in their cytosolic tails which bind to the tandem SH2 domain proteins Syk and phosphatidylinositol (PI) 3-kinase. This includes the GPVI-FcRγ complex and the low affinity immune receptor, FcγRIIA, which signal via an immuno-receptor-tyrosine-based activation motif (ITAM) characterised by two conserved YxxL sequences separated by 6–12 amino acids [3,4]. CLEC-2, on the other hand, signals via a single YxxL known as a hemITAM, and PEAR1 via a single YxxM sequence [2,5]. The binding of the tandem SH2 domains in Syk to phosphorylated ITAM or two hemITAM sequences initiates a signalling cascade that leads to activation of phospholipase C (PLC) γ2. The binding of the tandem SH2 domains in PI 3-kinase to two phosphorylated YxxM sequences leads to the activation of PI 3-kinase and generation of phosphatidylinositol 3,4,5-trisphosphate (PIP3) and recruitment of pleckstrin homology (PH) domain proteins to the membrane, including the kinases Akt and Btk.

In platelets, a net increase in phosphorylation of the cytosolic tails of glycoprotein receptors occurs when a sufficient density is reached for Src and Syk kinases to overcome the effect of active tyrosine phosphatases in the membrane such as CD148, SHP1 and SHP2 [6,7]. Multivalent ligands therefore induce activation through receptor clustering whereas monovalent ligands act as antagonists. In addition, ligand engagement may lead to a conformational change that increases the availability of tyrosines for phosphorylation, as exemplified by the dissociation of ITAMs from the inner leaflet of the membrane in the T cell receptor [8]. There is no evidence however that this plays a role in mediating activation of platelet glycoprotein receptors.

The reversible binding of a monovalent ligand to a receptor has been modelled by the law of mass action which states that the rate of a chemical reaction is proportional to the concentration of the reactants [9]. As originally proposed, the law of mass action describes receptor occupancy but not the conformational change that gives rise to the activation of multi-membrane spanning receptors such as GPCRs and ion channels. This was followed by the two-state model of receptor theory which accounts for this conformational change and provides a molecular explanation of efficacy [10]. More complex models of receptor activation have since emerged to account for additional features of receptors such as desensitisation and inverse agonists (for review see [11]).

In 2008, Cooper and Qian [12] applied the law of mass action to monomeric receptors which signal through Src family kinases. The ordinary differential equations (ODEs) developed in their study led them to conclude that phosphorylation of these receptors by Src kinases can occur solely as a result of receptor clustering without the need for a net change in kinase activity, receptor conformation or movement to a specialised location in the membrane such as a lipid raft. They proposed that clustering of receptors generates a critical density of receptor tyrosines for phosphorylation and binding of Src kinases via their SH2 domains, promoting transphosphorylation of the Src kinases and receptor phosphorylation thereby initiating downstream signalling [12]. However, they also emphasised that additional factors could play a role, such as other signalling proteins or the cytoskeleton. These additional interactions can be further developed using ODE-based models, but they rapidly become complex.

Agent-based modelling (ABM) is an alternative computational method that does not require a full understanding of all of the components of the system and can be used to simulate the actions and interactions of autonomous agents in order to identify key regulatory steps in a complex system [13, 14]. ABM also has the advantage that it enables spatial relationships to be considered, whereas this is not easily done for equation-based methods. ABM has been used in many areas, including in sociology, town-planning and economics, but has only been applied to the study of receptors in a few instances, with a primary focus on the modelling of complex intracellular signalling pathways [15, 16]. In ABM, it is relatively easy to incorporate rules that govern the clustering of receptors such as ligand binding, location, phosphorylation and interactions with other receptors and signalling molecules, and thereby study their interactions.

In the present study, we have used ODE and ABM to explore the reversible binding of soluble ligands (monovalent, divalent and tetravalent) and membrane-bound ligands (monovalent) to platelet glycoprotein receptors. For the ODE-based models, we have assumed that the receptors are monomers, but for the agent-based models, we have modelled receptors as a mixture of monomers and dimers, and introduced further complexity through a divalent cytosolic crosslinker to mimic the tandem SH2 domains of Syk and PI 3-kinase. We have tested the models by monitoring receptor activation in platelets and by measurement of receptor clustering in transfected cell lines using fluorescence correlation spectroscopy (FCS) in the presence of monovalent, divalent and multivalent ligands.

## Results

### Monomeric receptors can be clustered by a variety of ligands

**Fig 1:**
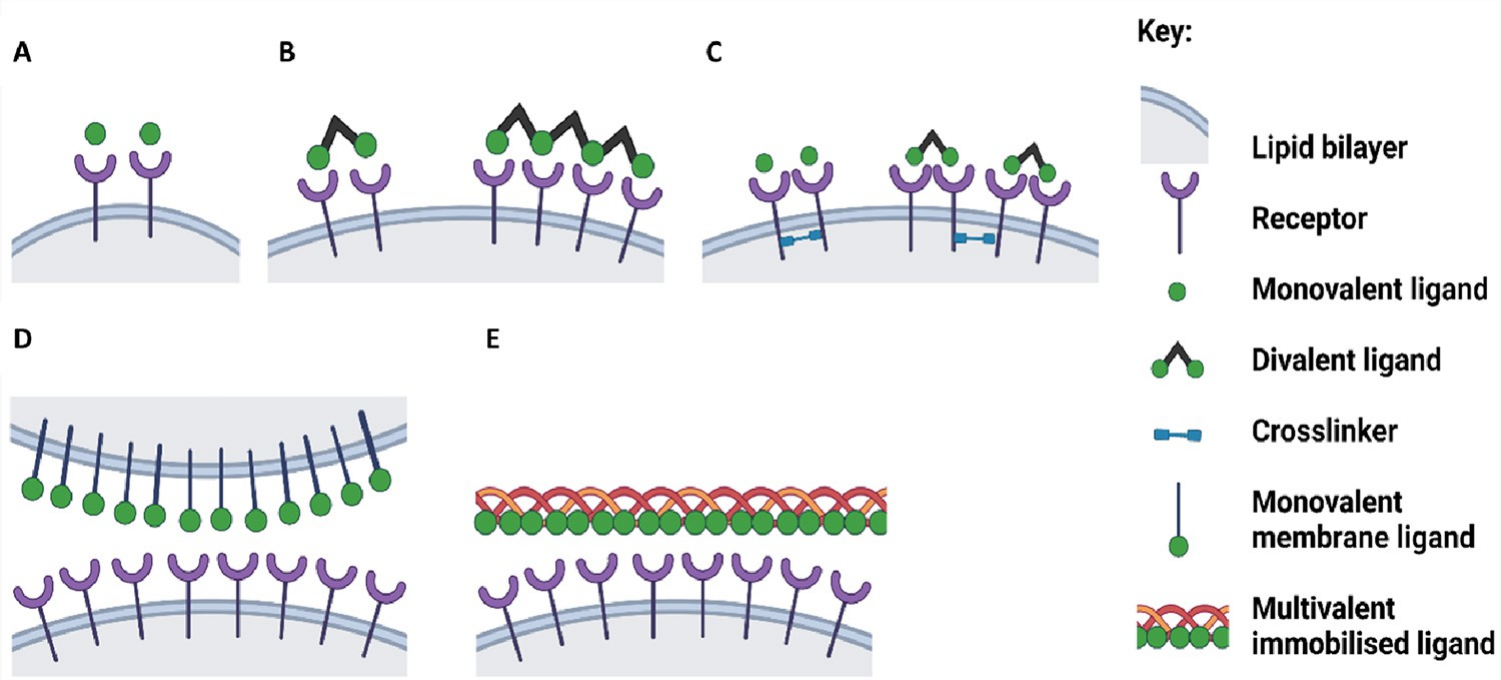
Schematic representation of ligand-receptor interactions. Representations of monovalent and multivalent ligand-receptor interactions: the receptor is shown as a monomer: (A) soluble monovalent ligand (B) soluble divalent and tetravalent ligands (C) soluble monovalent or divalent ligand and a cytosolic crosslinker (D) monovalent membrane ligand (E) multivalent immobilised ligand.

Fig 1 shows a representation of the interactions that govern the regulation of monomeric (i.e. receptors with one binding epitope) platelet glycoprotein receptors. The interaction of soluble monovalent, divalent and tetravalent ligands with a monomeric receptor are depicted in Fig 1A and 1B. Examples of monovalent ligands are an antibody Fab or a nanobody, and of a divalent ligand an antibody or dimerised nanobody. A tetravalent ligand can be generated by the crosslinking of monovalent ligands such as a nanobody, as generated in this study, and is representative of a multivalent ligand. Fig 1C illustrates how a tandem SH2 domain-containing protein, representing Syk or PI 3-kinase, can further support receptor clustering by causing cross-linkage of receptors in the cytoplasm. Fig 1D and 1E show a monovalent membrane ligand and an immobilised multivalent ligand, as exemplified by the CLEC-2 ligand podoplanin and the GPVI ligand collagen, respectively.

The law of mass action was introduced to model the interaction of a drug with its receptor by Clark in 1933 [9] with the following assumptions: (i) all reactions are reversible (ii) the interactions are one-to-one (iii) each interaction is an independent event (iv) the ligand concentration is in excess over that of the receptor such that the change in ligand concentration on binding to the receptor is negligible. These assumptions enabled the relationship between ligand concentration and response to be investigated and later, when radioligand binding studies were developed, the link to occupancy. The second and third assumptions are not valid, however, for glycoprotein receptors that are activated by clustering as receptor binding and activation are dependent on the combination of affinity and avidity, while the fourth assumption does not apply to surface-restricted ligands. Nevertheless, the principle that the interaction of a single epitope in a ligand with a monomeric receptor is determined by the law of mass action and that this is the basis of activation has been shown to be valid [12]. The present study further explores this relationship using ODEs and ABM as applied to ITAM, hemITAM and YxxM-containing receptors.

### ODE modelling

#### ODE modelling of the interaction of a monovalent ligand and a monomeric receptor

The interaction of a monovalent soluble ligand and a monomeric receptor was modelled using ODEs according to law of mass action [17]. At equilibrium, the law defines the amount of the ligand-receptor [LR] complex in relation to the concentration of ligand [L] and receptor [R] as shown in Equation (Eq) 1.1:

$$L+R\overset{k_{1}}{\underset{k_{-1}}{⇌}}LR\;.$$ (1.1)

The amount of LR is determined by the association (*k*_1_) and dissociation (*k*_-1_) rate constants, which have units of M^-1^s^-1^ and s^-1^_,_ respectively.

In the model by Clark [9], the concentration of the ligand is in vast excess of the receptor and it follows that, at equilibrium, the system reaches a steady state defined by the equilibrium dissociation constant, *K*_*D*_ (Eq 1.2). The unit of *K*_D_ is M, and *K*_D_ is given by:

$$K_{D}=\frac{k_{-1}}{k_{1}}=\frac{\left[L].[R\right]}{\left[\mathrm{LR}\right]}.$$ (1.2)

This may also be expressed as:

$$\frac{\left[\mathrm{LR}\right]}{R_{\mathrm{tot}}}=\frac{\left[L\right]}{K_{D}+[L]},$$ (1.3)

where R_tot_ denotes the total number of receptors. The reciprocal of *K*_*D*_ is the equilibrium association constant, *K*_*A*_, with units of M^-1^:

$$K_{A}=\frac{1}{K_{D}}.$$ (1.4)

For the model where the ligand is assumed to be in vast excess of the receptor, and therefore the concentration is not significantly reduced, the *K*_*D*_ is equivalent to the concentration of ligand required to occupy 50% of the receptors, with greater than 95% receptor occupancy occurring at a 19-fold higher concentration. The dynamics of the ligand-receptor interaction are defined by a linear ODE, whose solution gives the following standard result [18]:

$$[LR](t)=R_{tot}.\frac{\left[L\right]}{K_{D}+[L]}.(1-e^{-(k_{1}.[L]+k_{-1})t}).$$ (1.5)

**Fig 2:**
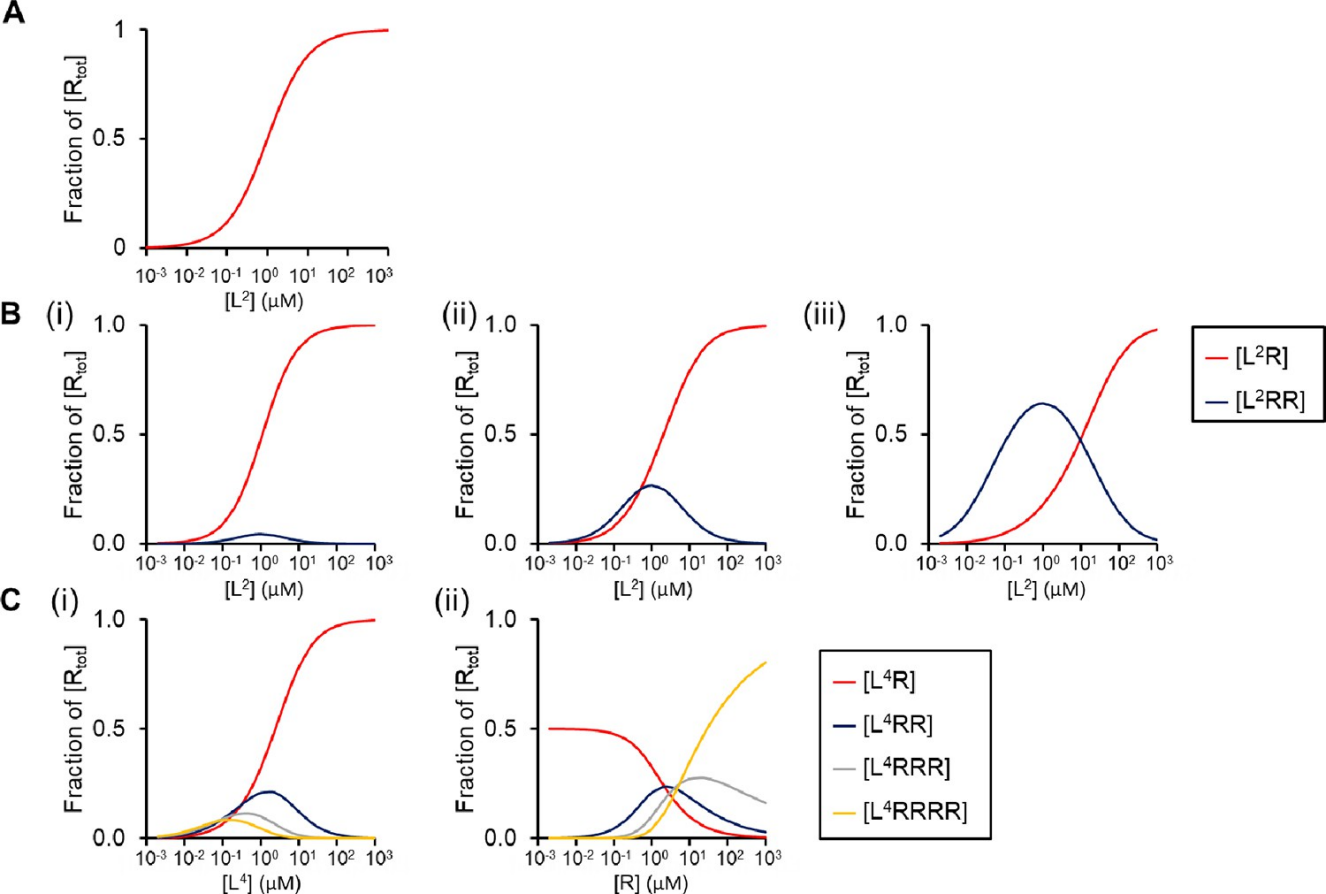
Interaction of monovalent, divalent and tetravalent ligands with a monomeric receptor. (A) The concentration-occupancy relationship at equilibrium of a monovalent ligand (L) and a monomeric receptor (R). The ligand is maintained at a constant concentration (red). The following parameters have been used: *K*_D_ = 1 μM, *k*_1_ = 1 μM^-1^s^-1^, *k*_-1_ = 1 s^-1^. (B) The concentration-occupancy relationship at equilibrium for a soluble divalent ligand [L^2^] and monomeric receptor, varying the second association rate constant (*k*_2_*)*: the following values have been used: k_1_ = 1 μM^-1^s^-1^, k_-1_ and k_-2_ = 1 s^-1^ (i) *k*_2_ = 0.1 μM^-1^s^-1^ (ii) *k*_2_ = 1 μM^-1^s^-1^ (iii) *k*_2_ = 10 μM^-1^s^-1^. L^2^R and L^2^RR reflects a divalent ligand bound to 1 and 2 receptors, respectively. (C) The concentration-occupancy relationship at equilibrium for a soluble tetravalent ligand (L^4^) and monomeric receptor varying the concentration of (i) ligand and (ii) receptor; the concentration of L^4^ in (ii) was 1 μM. The other parameters are: *k*_n_ = 1 μM^-1^s^-1^ and *k*_-n_ = 1 s^-1^. The receptor concentration in (A, B and C) is 1 μM. The graphs were generated with the MATLAB code: https://github.com/zeemaqsood/TAPAS_ESR9_Modelling_Project. The selected parameters are for illustrative purposes.

The relationship between ligand concentration and occupancy when plotted on a semi-log plot is sigmoid (Fig 2A).

The assumption that the ligand concentration is in excess over the receptor concentration does not apply to a transmembrane ligand since the concentration will reduce upon binding to the receptor, unless it can be *rapidly* replenished from other regions of the membrane or by fusion of intracellular membranes. Thus, the membrane density will influence the concentration-occupancy relationship. The consumption of the ligand would cause a shift to the right in the concentration response curve, with the size of the shift further increased by the reduction in the receptor concentration.

The time course of ligand binding to the receptor can also be modelled (Eq 1.5). For a soluble ligand, the ODE model shows that the time to equilibrium decreases with increasing ligand concentration, but is independent of the receptor level (S1A and S1B Fig). The stochastic nature of the interaction can be modelled using Gillespie’s algorithm [19]. The degree of occupation varies with the association and dissociation constants and receptor number, with a greater variation in occupancy with fewer receptors expected due to the increase in stochasticity (S1C Fig).

In summary, for a soluble monovalent ligand, the ODE modelling shows the relationship between ligand concentration, receptor occupancy and time, and how these vary in proportion to the association and dissociation constants and receptor number. The equilibrium receptor occupancy curve is approximately right-shifted when moving to the ligand-depletion model for a membrane-bound ligand.

### ODE modelling of the interaction of a divalent ligand and a monomeric receptor

We have used ODEs to model the interaction of a divalent ligand with a monomeric receptor. In the equations below the divalent ligand is depicted as L^2^ to reflect two epitopes and the receptor as R or RR to reflect a monomeric or dimeric receptor, respectively. There are two species of ligand-receptor combinations, L^2^R and L^2^RR. The introduction of a second epitope generates two equilibria:

$$L^{2}+R\overset{k_{1}}{\underset{k_{-1}}{⇌}}L^{2}R\;,$$ (2.1)

$$L^{2}R+R\overset{k_{2}}{\underset{k_{-2}}{⇌}}L^{2}RR\;,$$ (2.2)

with equilibrium dissociation constants, *K*_*D1*_ and *K*_*D2*_, respectively:

$$K_{D1}=\frac{k_{-1}}{k_{1}}=\frac{\left[L^{2}]\cdot [R\right]}{\left[L^{2}R\right]},$$ (2.3)

$$K_{D2}=\frac{k_{-2}}{k_{2}}=\frac{\left[L^{2}R]\cdot [R\right]}{\left[L^{2}\mathrm{RR}\right]}.$$ (2.4)

The two reactions (Eqs 2.1 and 2.2) have a shared intermediate species L^2^R acting as a product for Eq 2.1 and as a substrate for Eq 2.2. The relationship between the *K*_*D*_ values of coupled reactions 1 and 2 is multiplicative [20] and introduces a new constant *K*_*D3*_ which is the product of *K*_*D1*_ and *K*_*D2*_:

$$K_{D3}=K_{D1}{∙K}_{D2}.$$ (2.5)

The units of *K*_*D3*_ are M^2^, and so this constant is not equivalent to *K*_*D1*_ and *K*_*D2*_ which have units of M.

The binding of the first epitope in a soluble divalent receptor will influence the binding of the second epitope as a result of avidity, while dissociation from either receptor would return the dimeric receptor, L^2^RR, to L^2^R. A change in conformation of the divalent ligand could also influence the association and dissociation rate constants necessitating the introduction of an additional term, denoted by α, which would change the association and dissociation rate constants for the interconversion of L^2^R and L^2^RR:

$$k_{2}=\alpha_{+}∙k_{1}$$ (2.6)

$$k_{‐2}=\alpha_{-}∙k_{-1}.$$ (2.7)

The ratio of α_+_ and α_−_can be defined as a single variable, α, a cooperativity coefficient with a value of >1, equal to 1 and <1 denoting positive, neutral and negative cooperativity, respectively [21]:

$$\alpha =\frac{\alpha_{+}}{\alpha_{-}}.$$ (2.8)

The value of α consequently influences the *K*_*D2*_ value, which is given by:

$$K_{D2}=\frac{k_{-2}=\alpha_{-}\cdot k_{-1}}{k_{2}=\alpha_{+}\cdot k_{1}}=\frac{\left[L^{2}R]\cdot [R\right]}{\left[L^{2}\mathrm{RR}\right]}.$$ (2.9)

The concentration-response relationship for the formation of L^2^R and L^2^RR with increasing concentrations of L^2^ and its dependency on the association rate constant *k*_2_, while keeping the other rate constants the same, is shown in Fig 2B. For the purpose of modelling, the values of the dissociation rate constants *k-*_1_ and *k-*_2_ are the same as *k*_1_. The curves are generated by running the ODE solvers in Matlab to find the long-time solution. The curves can also be generated using an ODE model by solving a quadratic equation [22].

The amount of L^2^RR shows a bell-shaped relationship to the ligand concentration with the peak of L^2^RR occurring when the concentration of ligand is equal to *K*_D1_ and then declining as the ligand concentration increases, while the level of L^2^R continues to increase (Fig 2B). The magnitude of the peak in L^2^RR also increases with increasing values of *k*_2_. This is to be expected as this favours the formation of L^2^RR from L^2^R and reflects the influence of avidity. The modelling also shows that the ratio of L^2^R to L^2^RR is influenced by the receptor concentration, with a greater proportion of L^2^RR with increasing concentrations of the receptor. In other words, when the receptor concentration is low the probability of a divalent ligand finding a second receptor to form L^2^RR is reduced. The time to equilibrium also decreases as the concentration of ligand and receptors increases.

In summary, the introduction of a second epitope in the ligand leads to a further state, L^2^RR, and two new rate constants, *k*_*2*_ and *k*_*-*2_. This leads to a bell-shaped response curve for L^2^RR, which peaks when the concentration of ligand is equal to *K*_D1_ and with the size of the peak increasing in proportion to the forward rate constant, *k*_*2*_, while keeping all other rate constants the same. The amount of L^2^RR for a given ligand concentration also increases as the receptor number increases.

### ODE modelling of the interaction of a tetravalent ligand and a monomeric receptor

Many of the ligands of platelet glycoprotein receptors have more than two epitopes such as extracellular matrix proteins, snake venom proteins, multisulfated sugars and diesel exhaust particles [23]. We have used a tetravalent ligand (depicted as L^4^) as representative of a multivalent ligand and modelled this using ODEs. The interaction of the tetravalent ligand with a monomeric receptor will generate four ligand-receptor species: L^4^R, L^4^RR, L^4^RRR and L^4^RRRR, and therefore four equilibria defined by the order of attachment from unsaturated ligand to fully saturated ligand, with corresponding *K*_*D*_ values for each reaction (Eqs 3.1–3.5 in S1 Text). Fig 2C shows the effect of the ligand and receptor concentration on the occupancy curve at equilibrium with neutral cooperativity (α = 1). The relationships between the concentration of the ligand and receptor occupancy, other than for occupancy of a single receptor, L^4^R, are bell-shaped with L^4^R predominating at higher ligand concentrations (Fig 2C). In contrast, with increasing receptor concentrations L^4^RRRR will predominate for a fixed concentration of L^4^ (Fig 2C). Thus, as with a divalent ligand, the concentration of the ligand and the concentration of receptors are important in determining the stoichiometry of ligand-receptor binding as ligand valency increases.

### Experimental validation of ODE models

#### Tetravalent but not divalent nanobodies activate GPVI

The ODE models define the relationship between receptor occupancy and ligand valency. To test the models, functional studies were undertaken using novel nanobody-based monovalent, divalent and tetravalent ligands to the platelet collagen receptor GPVI based on the assumption that a higher ligand valency would increase affinity (as a consequence of avidity) and signal strength. The divalent and tetravalent ligands were generated by crosslinking of a high affinity nanobody, Nb2, that binds to the first immunoglobulin (Ig) domain in GPVI [24]. A flexible 15 amino acid linker (GGGGS)_3_ was used to generate a divalent ligand, Nb2-2, and a tetravalent ligand, Nb2-4, as shown in S2A Fig. Nb2-2 and Nb2-4 were expressed in bacteria and purified using a His-tag, showing a single major band of the expected size (S2B Fig).

**Fig 3:**
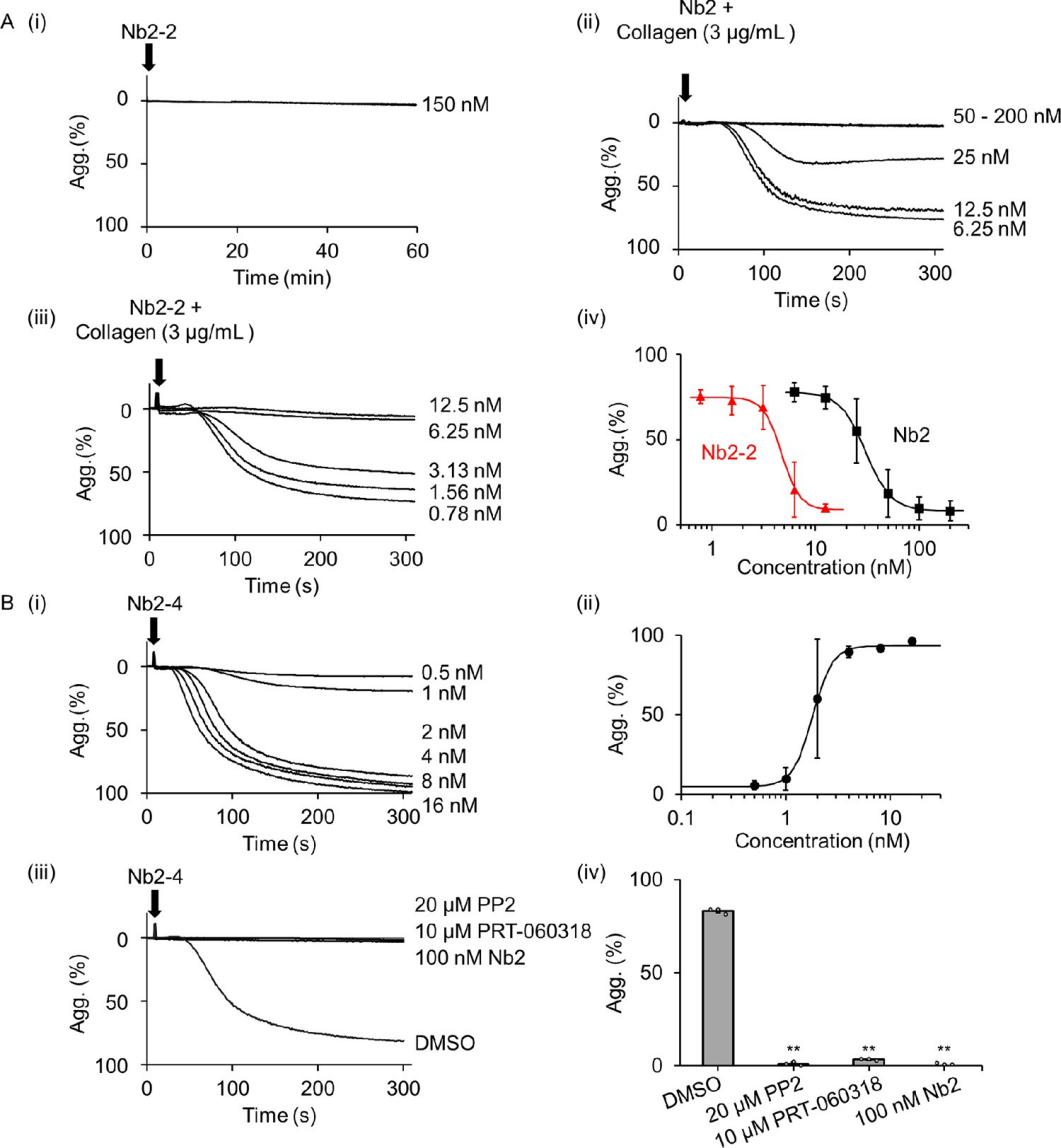
The effect of monovalent, divalent and tetravalent Nb2 on platelet aggregation. (A) Representative traces of platelets incubated with (i) Nb2-2 (ii) Nb2, 5 min before collagen (3 μg/mL) (iii) Nb2-2, 5 min before collagen (3 μg/mL); (iv) concentration-response relationship of inhibition of aggregation (Agg) to collagen by Nb2 and Nb2-2, results shown as mean + s.d. (B) (i) Representative traces of platelets stimulated by Nb2-4 (ii) concentration response curves with results shown as mean ± s.d. aggregation (%) after 5 min. (iii) Representative traces of platelets incubated with vehicle (0.1% DMSO), PP2 (20 μM), PRT-060318 (10 μM) or Nb2 (100 nM) for 5 min before stimulation with Nb2-4 (16 nM) (iv) histogram showing mean ± s.d. aggregation (%) after 5 min; all five treatment groups were compared using one way ANOVA analysis, followed by Tukey test: **(P < 0.01); n = 3.

Neither Nb2 nor Nb2-2 (150 nM) induce platelet aggregation when incubated for up to 60 min (Fig 3A[i]). On the other hand, Nb2 and Nb2-2 block aggregation to collagen (3 μg/mL) with IC50 values of 30.7±1.9 nM and of 4.7±0.5 nM, respectively (Fig 3A[ii-iv]). Nb2-2 also exhibited a 6-fold greater affinity (*K*_D_ = 0.100 + 0.003 nM) than Nb2 (*K*_D_ = 0.58 + 0.06 nM) when measured by surface plasmon resonance (SPR; S3 Fig and [24]). The greater potency and affinity of Nb2-2 relative to Nb-2 shows that both epitopes can bind to GPVI on platelets and on immobilised protein, respectively, with the increase in affinity being due to avidity.

Nb2-4 stimulated rapid aggregation of platelets with a maximal response at a low nanomolar concentration suggesting that it is able to crosslink at least 3 and possibly 4 GPVI receptors (Figs 3B[i-ii] and S2C [i,ii]). The increase in crosslinking accounts for its ability to induce sufficient clustering of GPVI to drive platelet aggregation. As expected, aggregation to Nb2-4 was blocked by monovalent Nb2 and by inhibitors of Src and Syk tyrosine kinases (Fig 3[iii-iv]) confirming that it was mediated through clustering of GPVI. The highest concentration of Nb2-4 tested (100 nM) induced full platelet aggregation (Fig 3B[ii]) with no evidence of a bell-shaped concentration response relationship as seen in the ODE model. The affinity of Nb2-4 for GPVI measured by SPR was similar to that of Nb2-2 (S3 Fig).

These results demonstrate that a tetravalent but not a divalent ligand is able to activate platelets through GPVI and that a divalent ligand has a greater potency than a monovalent ligand for inhibition of platelet activation by collagen.

### Agent-based modelling

#### Agent-based modelling to monitor ligand binding and cluster formation

**Fig 4:**
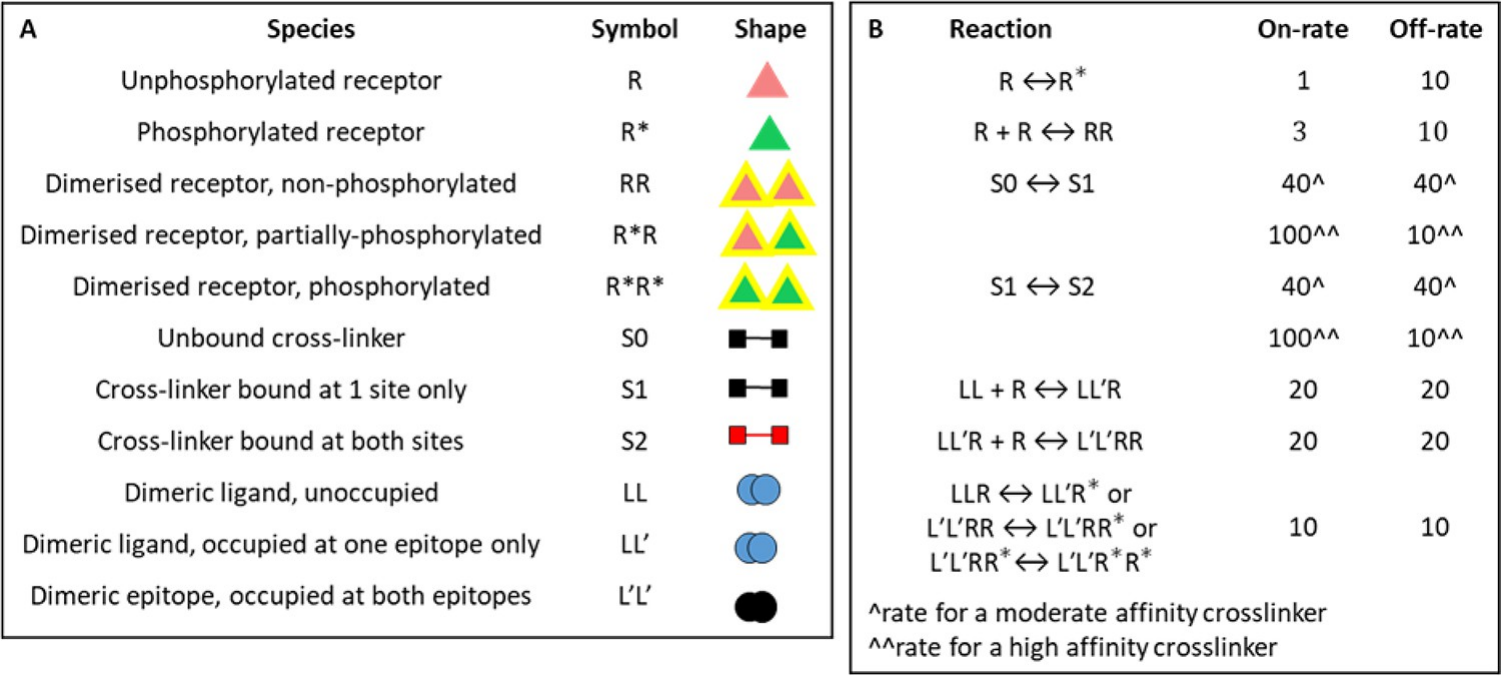
Parameter values and symbols used for agent-based modelling. **(A)** The symbols used to represent species in the agent-based model Figures are shown. The number of receptors (R), crosslinkers (S) and divalent ligands (LL) per box is 100 unless stated. **(B)** The default values for on-rates and off-rates are shown in ticks (arbitrary unit of time), with one tick corresponding to the ‘go’ function within each run. These represent the *probability* of a reaction to occur out of 100 runs. These are arbitrary values selected for illustrative purposes. They have been chosen to give a low basal level of dimerisation and phosphorylation to correspond to a resting platelet. Two sets of values have been chosen to reflect a moderate and high affinity crosslinker in regulating cluster formation; the latter takes into account that the affinity of a ‘moderate’ affinity crosslinker can be increased by its co-localisation in the membrane as is the case for the majority of SH2-domain containing proteins. A ‘low’ value was selected for the affinity of the ligand to reflect the affinity of individual epitopes in endogenous ligands for platelet glycoprotein receptors, noting that this can be increased by avidity. The selection of a relatively low value enables illustration of how variables can combine to drive clustering. The rate of movement of receptors is constant at 1 patch per tick patch is an arbitrary unit of area (there are 441 patches per box). The default values give rise to to a basal value of 10% receptor dimerisation and 10% receptor phosphorylation in the absence of crosslinker. The crosslinker can only bind to a phosphorylated receptor: the probability of each epitope of the crosslinker to bind to a phosphorylated receptor is shown as an average frequency per 100 ticks. Assuming that both epitopes are occupied, the ‘moderate’ affinity crosslinker will remain bound to two receptors for 16 out of 100 ticks on average. The ‘high’ affinity crosslinker will remain bound to the receptor for 81 out of 100 ticks, on average. The probability of each epitope of the divalent ligand to attach to a receptor upon collision was set to 20 out of 100 ticks and to dissociate to 20 out of 100 ticks. Assuming that both epitopes of the divalent ligand are bound at the same time, a fully occupied divalent ligand will remain bound to both receptors for an average 4 out of 100 ticks.

We have developed an agent-based model in NetLogo [25] to complement the modelling of ligand-receptor interactions using ODEs as this can be more easily developed to include greater complexity such as receptor homo-dimerisation and a cytosolic crosslinker to mimic a tandem SH2 domain protein. In addition, ABM incorporates spatial interactions which is particularly relevant to clustering of monomeric receptors. The symbols of the agent-based models and default values for on- and off-rates are shown in Fig 4A. All reactions were run on 30 occasions and results are shown as mean + s.d. by which time equilibrium had been reached.

For the agent-based model, the following assumptions apply: (i) the crosslinker is only able to bind to a phosphorylated receptor (ii) the mobility of monomeric and dimeric receptors is the same (iii) all reactions are reversible (iv) the speed of a cluster (> 3 receptors) is inversely proportional to its size (v) the binding of the cytosolic crosslinker requires receptor phosphorylation and protects against dephosphorylation. Receptor clustering can be achieved by a combination of (i) receptor-receptor homo-dimerisation (ii) binding of a cytosolic crosslinking protein (iii) a divalent or multivalent ligand. Together, these can lead to formation of receptor trimers, tetramers and higher order oligomers. The assumption that the binding of the cytosolic crosslinker requires receptor phosphorylation and protects against dephosphorylation reflects the situation in a cell, as shown using phosphorylated peptides [26]. The reduction in diffusion of large clusters was introduced as they diffuse at a slower rate in the membrane [27], but when tested in a limited number of scenarios, removal of this parameter did not have a significant effect on protein phosphorylation or receptor clustering.

We have systematically studied each of these parameters and show their effect on receptor dimerisation, receptor clustering and receptor phosphorylation.

#### Agent-based modelling of receptor dimerisation in the absence of a ligand or crosslinker

We first used ABM to model dimerisation of a receptor that undergoes reversible dimerisation in the absence of ligand or a crosslinker (Eq 4.1):

$$R+R\overset{k_{1}}{\underset{k_{-1}}{⇌}}RR\;.$$ (4.1)

**Fig 5:**
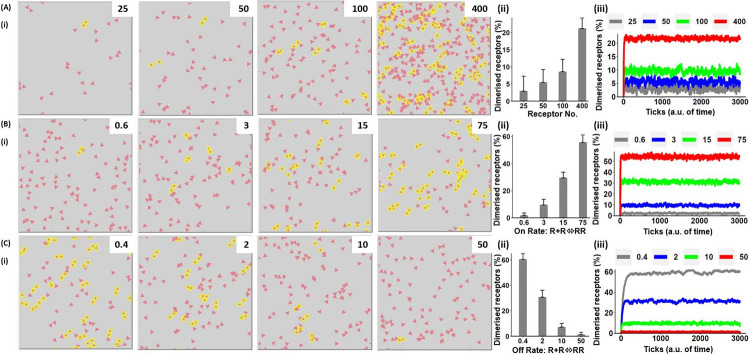
An agent-based model showing that the degree of receptor dimerisation increases with receptor number and rate of association. The effect of receptor number and rates of association and dissociation on receptor dimerisation (Eq 4.1) was modelled using an agent-based model. Unless stated, the parameter values and key are as described in Fig 4. (A) The effect of receptor number on formation of receptor dimers (i) representative runs at steady-state with the number of receptors shown in the upper right-hand corner (ii) the level of receptor dimerisation at steady-state (iii) the time course of receptor dimerisation. Results are shown as mean + s.d. of 30 simulations at 3,000 ticks. (B) The effect of varying the rate of receptor dimerisation (i) the rate of association is shown in the upper right-hand corner, for further details see part (A). (C) The effect of varying the off-rate of receptor dimerisation (i) the off-rate of association is shown in the upper right-hand corner, for further details see part (A).

The model shows that the degree of dimerisation is influenced by the receptor number and the rates of association and dissociation. As the receptor number increases, the number of collisions between receptors increases leading to an increase in the proportion of dimers increases (Fig 5A[i-iii] and S1 Video). Increasing the rate of association or decreasing the rate of dissociation also increases the number of receptor dimers (Fig 5B and 5C[i-iii]).

#### Agent-based modelling of receptor dimerisation in the presence of a divalent crosslinker

Introducing a divalent crosslinker that undergoes reversible binding to a phosphorylated receptor provides an alternative mechanism of clustering. In the agent-based model, the binding of the crosslinker prevents receptor dephosphorylation leading to a net increase in phosphorylation. This mimics the effect of binding a SH2 domain to a phosphorylated tyrosine in a cell. The effect of the crosslinker was modelled with on- and off-rates of receptor phosphorylation set to achieve 10% basal phosphorylation at the initiation of the run. This level of phosphorylation was selected to reflect the low level of receptor phosphorylation in a resting cell. The model was run in the presence of a ‘moderate’ or a ‘high’ affinity crosslinker as defined using the parameters in Fig 4.

**Fig 6:**
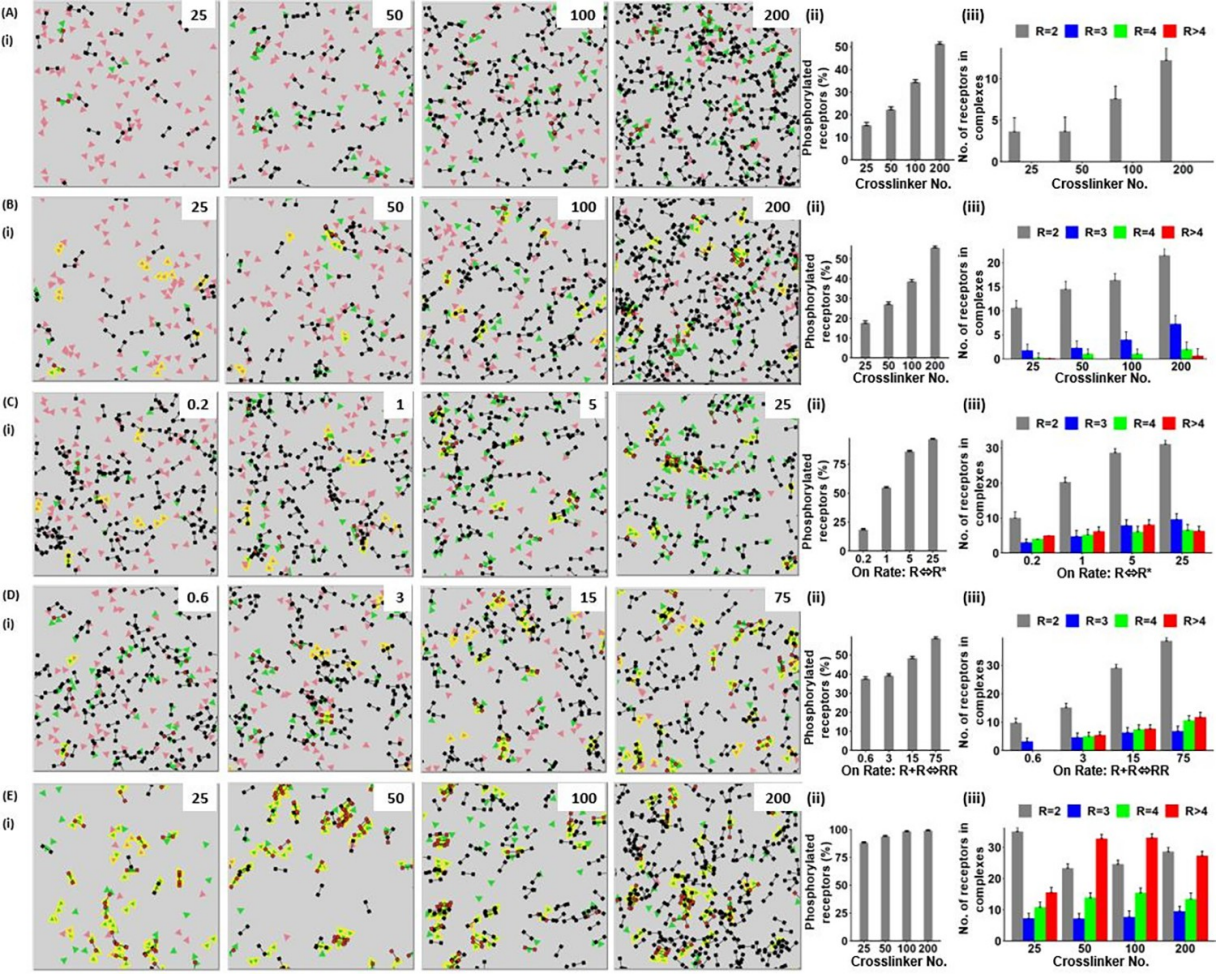
An agent-based model showing the clustering of receptors in the presence of a high affinity crosslinker. Agent-based modelling of the effect of changing the basal level of receptor phosphorylation and dimerisation, and the number of a high affinity crosslinker on receptor clustering. Unless stated, the parameter values and key are as described in Fig 4. (A) The effect of the number of high affinity crosslinkers on the proportion of receptor dimers for a receptor that is unable to dimerise (monomeric receptor) (i) representative runs at steady-state, the number of high affinity crosslinkers is shown in the upper right-hand corner (ii) number of receptors that are phosphorylated at steady-state (iii) number of receptor dimers (R = 2), trimers (R = 3), tetramers (R = 4) and higher order structures (R>4) at steady-state. Results are shown as mean + s.d. of 30 simulations at 3,000 ticks. (B) The effect of the number of high affinity crosslinker on clustering of a receptor with a basal level of dimerisation (10%) (i) the number of crosslinkers is shown in the upper right-hand corner, for further details see (A). (C) The effect of the basal level of receptor phosphorylation on clustering of receptors in the presence of a high affinity crosslinker (100 per box) (i) the rate of receptor phosphorylation per box is shown in ticks in the upper right-hand corner, for further details see (A). (D) The effect of the basal level of receptor dimerisation on clustering of receptors in the presence of a high affinity crosslinker (100 per box) (i) the rate of receptor dimerisation is shown in ticks in the upper right-hand corner, for further details see (A). (E) The effect of the number of high affinity crosslinkers on clustering of receptors in the presence of a high basal level of receptor dimerisation and phosphorylation. The rate of phosphorylation and degree of dimerisation are set to the highest values in (C) and (D) achieving ~80% and ~75% phosphorylation and dimerisation, respectively (i) The number of high affinity crosslinkers is shown in the upper right-hand corner, for further details see (A).

The model was first run with a monomeric receptor that can only be dimerised through binding of the crosslinker. There was a negligible change in the level of receptor dimerisation and phosphorylation in the presence of a moderate affinity crosslinker (S4A[i-iii) Fig]), whereas the presence of a high affinity crosslinker led to an increase in phosphorylation to over 50% and a doubling in the number of receptor dimers. There was no formation of higher order clusters, as expected, as dimerisation of the receptor monomers can only occur through binding of the crosslinker (Fig 6A[i-iii] and S2 Video).

The model was then run for a receptor that is able to undergo reversible dimerisation. The basal value of dimerisation was set at 10% in the absence of crosslinker (Fig 4). The introduction of a moderate affinity crosslinker resulted in a small increase in the number of receptor dimers and doubling of receptor phosphorylation at the highest crosslinker concentration, but there was negligible formation of higher order clusters (S4B[i-iii] Fig and S3 Video). The presence of a high affinity crosslinker led to an increase in the number of receptor dimers and the degree of receptor phosphorylation (both values reaching over 50%) and a small increase in the number of receptor trimers and tetramers (Fig 6B[i-iii]). The increase in the number of dimers, trimers and tetramers reflects the crosslinking of receptor monomers and dimers to themselves and to each other.

The model was extended to investigate the effect of increasing the basal level of receptor phosphorylation and receptor dimerisation. For these studies, the number of crosslinkers was set to the default parameter of 100 per box. The 1: 1 ratio of receptors to crosslinker is similar to the ratio of CLEC-2 and Syk in human platelets measured by quantitative proteomics (S1 Table). For comparison, S1 Table also shows the levels of these proteins in mouse platelets, as well as the levels of FcγRIIA, GPVI and PEAR1 in human and mouse platelets.

Increasing the rate of receptor phosphorylation had a negligible effect on the number of dimers and trimers in the presence of a moderate affinity crosslinker (S4C[i-iii] Fig). In contrast, in the presence of a high affinity crosslinker over 50% of receptors were present as dimer, trimers, tetramers and higher order oligomers (Fig 6C[i-iii] and S4 Video). A similar set of observations was seen when the basal level of receptor dimerisation was increased in the presence of a moderate or high affinity crosslinker (Figs S4D[i-iii] and 6[i-iii] and S5 Video). The combination of the increase in rate of phosphorylation and degree of dimerisation in the presence of a high but not moderate affinity crosslinker, led to a marked increase in the number and size of receptor clusters, with over 30% of receptors present in complexes of four, or great than four, when the number of crosslinkers was 50 or higher (Figs S4E[i-iii] and 6E[i-iii]).

These results show that receptor dimerisation, phosphorylation and crosslinker affinity regulate the degree of clustering, and that in combination they can lead to significant levels of clustering.

#### Agent-based modelling of receptor dimerisation in the presence of a divalent ligand

The readouts of the agent-based models are receptor clustering and phosphorylation, neither of which are altered in the presence of a monovalent ligand. For this reason, the models would not change in the presence of a monovalent ligand.

We have therefore used agent-based model to investigate the effect of a divalent ligand mimicking for example an antibody or dimerised nanobody. As this is similar to modelling the effect of a divalent crosslinker with a high basal level of receptor phosphorylation, as shown in Fig 6D, the modelling has only been carried out in combination with a divalent crosslinker. Low basal values of receptor dimerisation and phosphorylation were used, along with a moderate affinity crosslinker, in anticipation that the combination of these parameters may lead to synergy.

**Fig 7:**
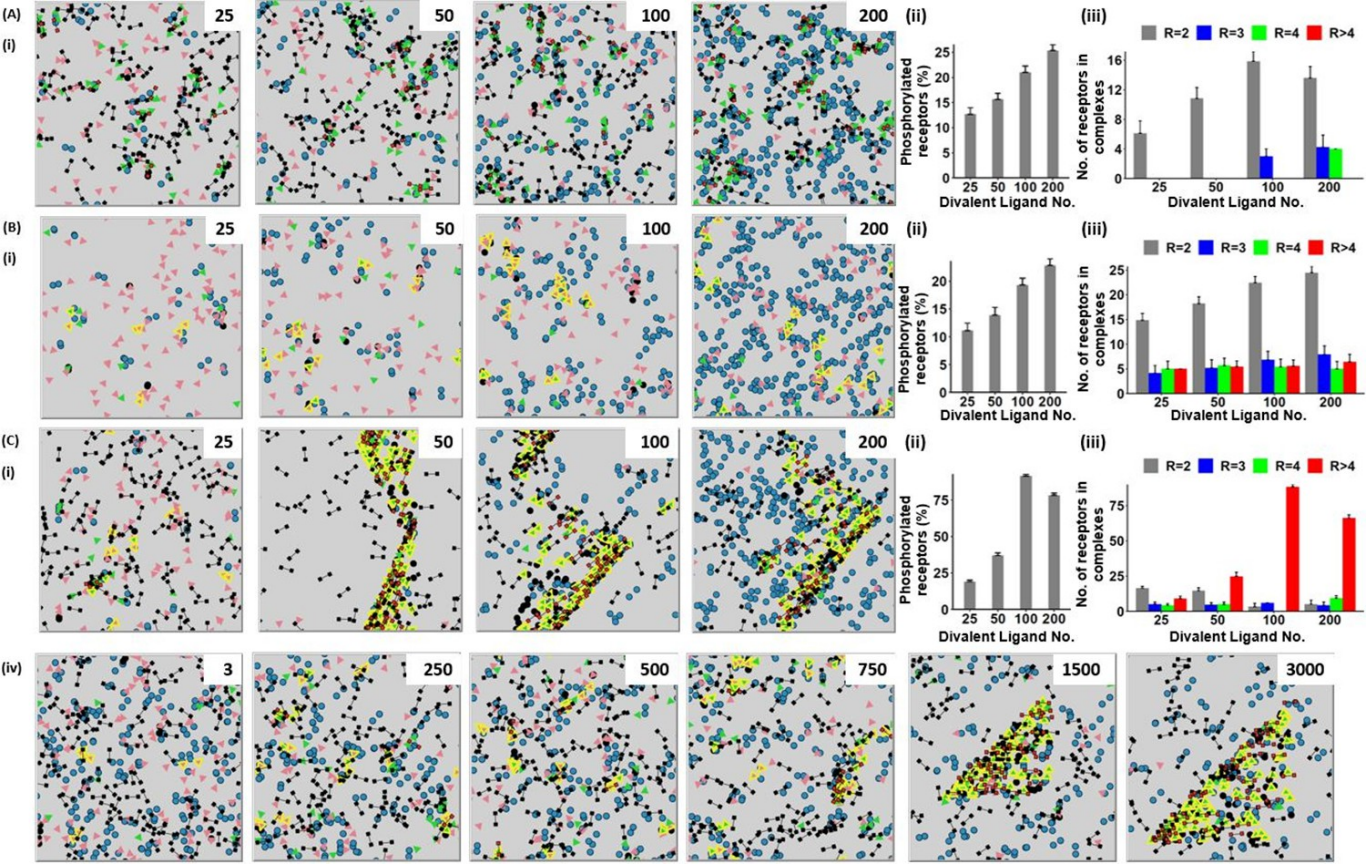
An agent-based model showing that the effect of a divalent ligand on receptor dimerisation and higher order clustering. Agent-based modelling was used to study the combination of a divalent ligand, receptor dimerisation and a cytosolic crosslinker on receptor clustering. Unless stated, the parameter values and key are as described in Fig 4. (A) The effect of a divalent ligand on clustering of a receptor that is unable to dimerise in the presence of a moderate affinity crosslinker (i) representative runs at steady-state, the number of divalent ligands is varied from 25–200 as shown in the upper right-hand corner (ii) number of receptors that are phosphorylated at steady-state (iii) number of receptor dimers (R = 2), trimers (R = 3), tetramers (R = 4) and higher order (R>4) structures at steady-state. Results are shown as mean + s.d. of 30 simulations at 3,000 ticks. (B) The effect of a divalent ligand on clustering of receptors with a low basal level of dimerisation (10%) (i) the number of divalent ligands is in the upper right-hand corner, for further details see (A). (C) (i-iii) The effect of a divalent ligand and moderate affinity crosslinking on clustering of receptors with a low basal level of dimerisation (10%) (i) the number of divalent ligands in the upper right-hand corner (iv) representative runs showing the time course of receptor clustering with the tick intervals are shown in the upper right-hand corner; the number of divalent ligands is 200, for further details see (A).

We first modelled the effect of a divalent ligand with a monomeric receptor that is unable to dimerise in the presence of a moderate affinity crosslinker and a basal level of receptor phosphorylation of 10%. This led to an approximate doubling in receptor phosphorylation and receptor dimerisation and formation of a small number of trimers and tetramers at the highest number of ligands (Fig 7A[i-iii]). In the presence of a low basal level of receptor dimerisation and absence of crosslinker, the divalent ligand led to a similar increase in receptor phosphorylation along with the formation of receptor trimers, tetramers and higher order oligomers even in the presence of a low ligand number (Fig 7B[i-iii]). Strikingly, the further addition of the moderate affinity crosslinker led to the formation of large receptor clusters which in some runs resulted in the formation of a single cluster (Fig 7C[i-iii] and 7[iv] and S6 Video).

This analysis shows that a divalent ligand can induce formation of a large receptor clusters in the presence of a low basal of receptor dimerisation and moderate affinity crosslinker. The relatively low nature of these values would help to minimise constitutive signalling in a cell whilst enabling rapid amplification upon ligand engagement.

### Experimental validation of the agent-based models

#### The effect of a Syk inhibitor on clustering of CLEC-2 and GPVI

The agent-based model in Fig 7C shows how the combination of a divalent ligand, receptor dimerisation, and crosslinker can drive receptor clustering. This model is applicable to CLEC-2 and PEAR1 whose phosphorylated tails are crosslinked by the tandem SH2 domains in Syk and PI 3-kinase, respectively. This is in contrast to GPVI where the tandem SH2 domains in Syk are believed to bind to two phosphorylated tyrosines in the FcR γ-chain ITAM.

We used fluorescence correlation spectroscopy (FCS) to investigate a role for Syk in the clustering of CLEC-2 and GPVI. FCS measures the fluorescence fluctuations brought about by the diffusion of fluorescently-tagged proteins through a small volume (~0.2 fl) in the membrane, generating information on the rate of diffusion and molecular brightness. The change in diffusion is relatively insensitive to cluster formation as an 8-fold change in mass results in a 1.6-fold change in diffusion coefficient [27]. In contrast, when used with photon counting histogram (PCH) analysis, FCS can be used to differentiate monomers, dimers and higher order oligomers [28].

**Fig 8:**
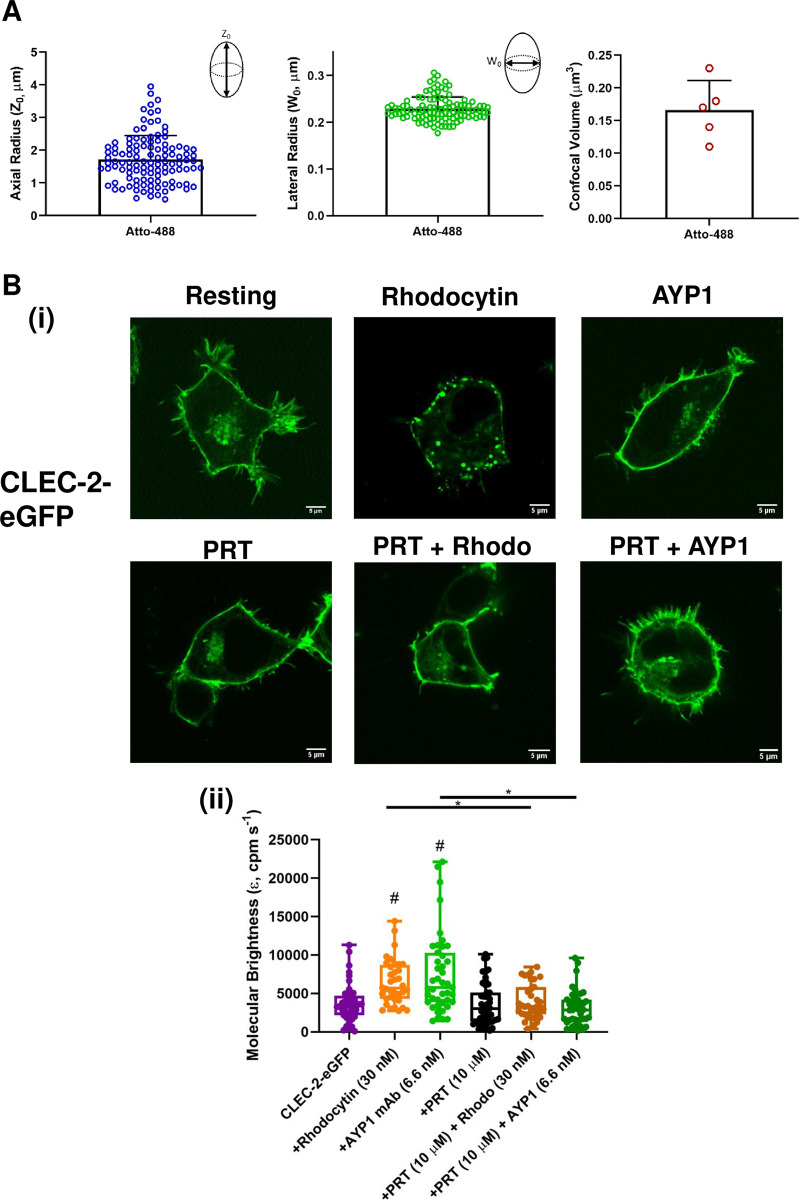
The Syk inhibitor PRT-060318 inhibits rhodocytin-and AYP1-induced CLEC-2 clustering. (A) Confocal microscope calibration using Atto-488 dye (10 nM) in water at 25°C. The axial (Z_o_) and lateral (W_o_) radii were determined to be ~1.72+0.72 μm and ~0.23+0.03 μm respectively and the confocal volume was ~0.17+0.05 μm^3^. > 110 FCS calibration measurements were taken in five experiments. Data are presented as mean ± s.d. (n = 5). **(B)** (i) Representative confocal microscopy images showing membrane localisation of CLEC-2-eGFP in resting and stimulated cells (field of view = 52 x 52 μm) (scale bar = 5 μm). (Bii) Box plots showing the molecular brightness (cpm s^-1^) of CLEC-2-eGFP, centre lines represent median, box limits indicate the 25^th^ and 75^th^ percentiles, and whiskers extend to minimum and maximum points. Concentration of rhodocytin (30 nM), mAb AYP1 (6.6 nM), PRT (10 μM) were used. Significance was measured with Kruskal-Wallis with Dunn’s *post-hoc* where *P* ≤ 0.05. In (ii) # = significance compared to CLEC-2 alone. FCS measurements were taken in 35–48 cells; n = 3–5.

To investigate the role of Syk in the clustering of CLEC-2, we expressed a N-terminal eGFP-tagged CLEC-2 in HEK293T cells and stimulated the cells with the snake venom toxin rhodocytin and the monoclonal antibody (mAb) to CLEC-2, AYP1. FCS measurements were carried out in the absence and presence of the Syk inhibitor, PRT-060318, which blocks phosphorylation of the hemITAM in CLEC-2 [29]. The membrane localisation of the eGFP-tagged CLEC-2 was verified by confocal microscopy (Fig 8B[i]).

Rhodocytin and mAb AYP1 induce clustering of CLEC-2 on the surface, as shown by confocal microscopy, which are blocked in the presence of PRT-060318 (Fig 8B[i]). In line with this, both ligands increase the molecular brightness of CLEC-2 as measured by FCS and this is also blocked in the presence of PRT-060318 (Fig 8B[ii-iii]) confirming a critical role of Syk in clustering of CLEC-2.

In contrast, PRT-060318 had no effect on clustering of C-terminal eGFP-tagged GPVI measured by confocal microscopy and FCS upon activation by a collagen-related peptide (CRP) or by the divalent and tetravalent nanobodies, Nb2-2 and Nb2-4 (S5A and S5B[i,ii] Fig). This is consistent with the tandem SH2 domains in Syk binding to the phosphorylated ITAM and not crosslinking adjacent receptors.

#### Synergistic action of a blocking F(ab)_2_ and Syk inhibitor in blocking platelet activation

**Fig 9:**
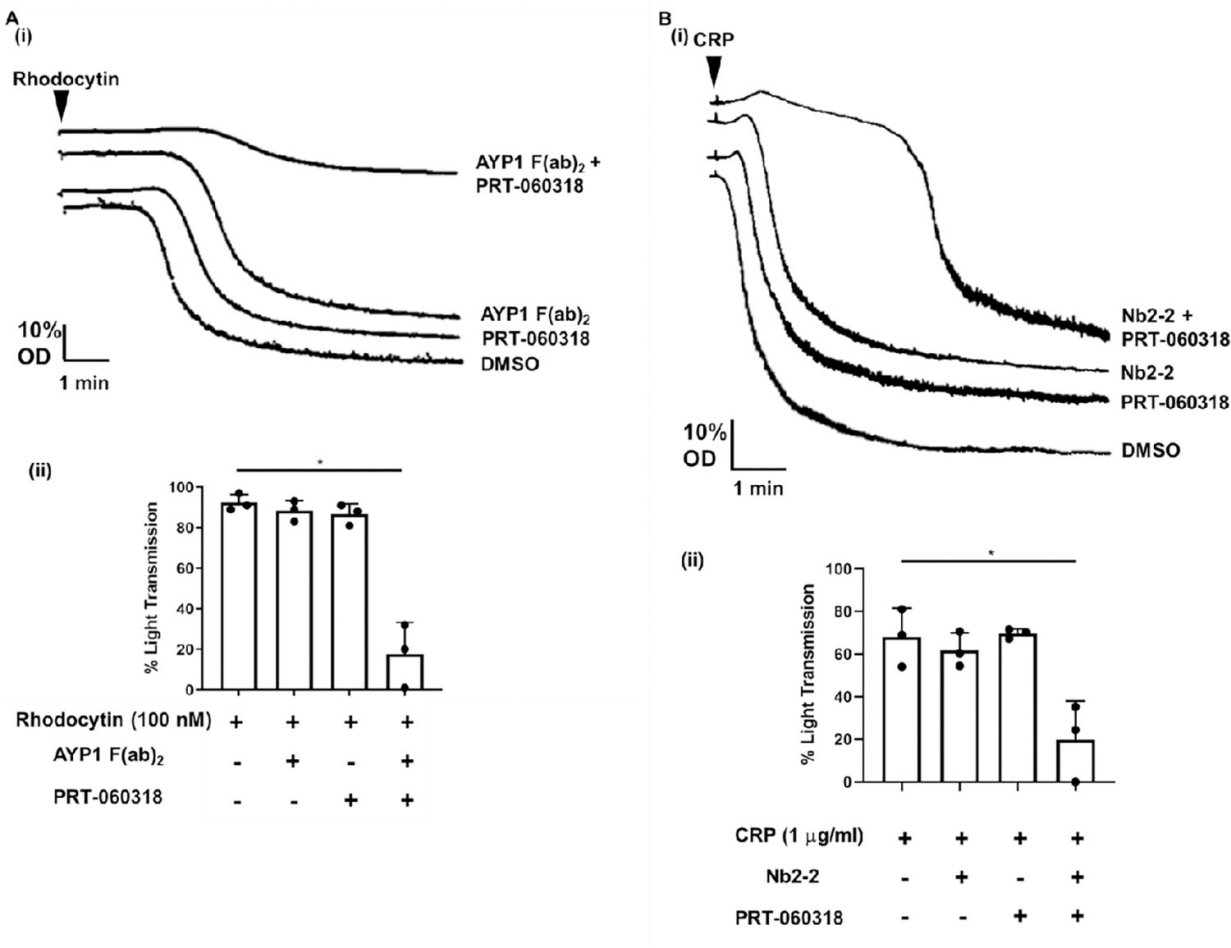
Synergistic inhibition of platelet aggregation by CLEC-2 and GPVI agonists by threshold concentrations of blocking biologics and the Syk inhibitor PRT-060318. (Ai) Representative traces of washed platelets (2x10^8^ platelets/ml) preincubated for 10 min with threshold concentrations of PRT-060318 (2.5 nM) and/or the divalent anti-CLEC-2 antibody fragment, AYP1 F(ab)_2_ (2.2 nM). The arrow indicates the time of addition of rhodocytin (100nM). (Aii) The mean ± s.d. level of aggregation (%) after 10 min (n = 3). (Bi) Representative traces of washed platelets (2x10^8^ platelets/ml) preincubated for 10 min with non-inhibitory concentrations of PRT-060318 (2.5 nM) and/or the divalent anti-GPVI nanobody, Nb2-2 (2.2 nM). The arrow indicates the time of addition of CRP (1 μg/ml). (Bii) The mean ± s.d. level of aggregation (%) after 3 min (n = 3: in one donor, 0.50 nM Nb2-2 was used due to 2.2 nM causing complete inhibition). Significance was measured using one-way ANOVA with a Tukey *post-hoc* test where *P ≤* 0.05.

The demonstration that clustering is regulated by binding of a divalent ligand and by a moderate affinity crosslinker suggests that blocking both pathways may have a synergistic effect. To investigate this, we tested the combination of a CLEC-2-blocking antibody fragment, AYP1 F(ab)_2_ (2.2 nM), and the Syk inhibitor, PRT-060318 (2.5 nM), at concentrations that have a minor/negligible effect on the response to rhodocytin. In combination, they caused a marked inhibition of the response to rhodocytin (Fig 9A[i,ii]).

To investigate if the inhibition could also reflect loss of signalling by Syk, we performed a similar study with the GPVI blocking nanobody, Nb2-2, and PRT-060318 (2.5 nM) against CRP (1 μg/ml). Nb2-2 has a similar subnanomolar affinity for GPVI to that of AYP1 (Fab_2_) for CLEC-2, and was used at the same concentration (2.2 nM) except in one donor whose platelets exhibited a much greater sensitivity to inhibition. The combination of the two inhibitors caused a marked inhibition of response to CRP (1 μg/ml), although in some experiments this resulted in a delay rather than abolition of aggregation, with a maximal response observed at later times (Fig 9B[i,ii]). This was not seen in the studies with rhodocytin.

## Discussion

The binding of multivalent ligands to platelet glycoprotein receptors such as CLEC-2, FcγRIIA, GPVI and PEAR1 leads to dimerisation and higher order clustering which drives formation of a critical density of tyrosine-based signalling motifs in the inner leaflet of the membrane for phosphorylation by active, membrane-associated kinases. This results in the binding of SH2 domains thereby overcoming the effect of active membrane phosphatases. In the present study, we have used ODE and ABM to model the interaction of these receptors with their ligands on the assumption that the interaction is driven by the law of mass action and that additional clustering can be achieved through receptor homo-dimerisation and a tandem SH2 domain protein.

In the ODE models, we have assumed that each epitope in a ligand binds to one receptor, that the concentration of the ligand is in vast excess of the receptor (unless stated), and that receptors are monomers. The ODE models show a bell-shaped concentration-response relationship for binding of divalent and tetravalent ligands (the latter representing multivalent ligands). In the ODE models, the divalent ligands binds to mixture of monomers dimers, and the tetravalent ligands to mixture of monomers, dimers, trimers and tetramers. Because of the effect of avidity, divalent and tetravalent ligands have a greater affinity for the receptor, as illustrated by SPR measurements for the divalent and tetravalent nanobody ligands to GPVI relative to the monovalent backbone. Further, confocal microscopy and FCS studies show that the two multivalent ligands but not the monovalent backbone cluster GPVI in a transfected cell line, thereby showing that they can crosslink GPVI on a cell surface. Strikingly, however, Nb2 and Nb2-2 are antagonists of collagen-induced activation of GPVI in human platelets, whereas tetravalent Nb2-4 induces powerful platelet aggregation. This suggests that four and possibly as low as three epitopes in a ligand are required for sufficient clustering of GPVI to drive platelet activation.

The concentration-response curve to Nb2-4 did not exhibit a bell-shaped relationship at higher concentrations in contrast to the ODE models. This is likely to be due to a combinaton of factors including (i) the stochastic nature of the ligand-receptor interaction, (ii) full receptor occupancy is not required for maximal aggregation by GPVI as shown by stimulation of platelets by CRP [30,31] and (iii) GPVI is expressed as a mixture of monomers and dimers as a consequence of homo-dimerisation and co-location on the surface [32]. The presence of dimers will lead to larger receptor complexes through crosslinking of monomers-to-dimers and dimers-to-dimers. While the presence of dimers can be modelled using ODE, this rapidly becomes complex and this does not cover co-association by chance. On the other hand, spatial consideration can be readily modelled by ABM, as well as other factors including receptor dimerisation and a cytosolic crosslinker (mimicking a tandem SH2 domain protein).

The agent-based model developed in this study shows how the presence of a divalent ligand, receptor homo-dimerisation and cytosolic crosslinker (and receptor phosphorylation) can work in combination to generate large receptor clusters. The importance of the crosslinker in CLEC-2 signalling was verified experimentally using confocal microscopy and FCS using an inhibitor of Syk which blocks phosphorylation of the CLEC-2 hemITAM [29]. Syk has a two SH2 domains which crosslink two phosphorylated CLEC-2 receptors. The agent-based model also shows a correlation between receptor density and receptor dimerisation, which may explain the increase in NFAT signalling that is observed with increasing GPVI concentrations in a transfected DT40 B cell line in the absence of a ligand, as this is predicted to increase receptor dimerisation [33]. This indicates that dimerisation of GPVI is sufficient to drive signalling. In contrast, the divalent nanobody against GPVI, Nb2-2, was an antagonist in human platelets. Similarly, an antibody to FcγRIIA, mAb IV.3, is an antagonist in human platelets [34]. The difference between the transfected cell line model and platelets may be related to the the level of GPVI expression and/or the levels of active kinases and phosphatases in the membrane, as well as differences in the sensitivity of the two assays. In platelets, there is a high level of basal tyrsoine phosphatase activity, as shown by the rapid increase in tyrosine phosphorylation in the presence of the phosphatase inhibitor peroxovanadate [35], and this helps to minimise activation of ITAM and YxxM receptors in the absence of a strong activating ligand. The importance of receptor diffusion in supporting receptor dimerisation and higher order clustering accounts is likely to account for the delay in onset of platelet activation by multivalent ligands of CLEC-2 [36], GPVI [37] and FcγRIIA [38, 39].

The ODE model was also applied to the interaction of a membrane or immobilised ligand. In both cases, the binding of the ligand to a receptor leads to a reduction in its concentration and thus a shift in the concentration-response curve to the right, although in reality this is more than countered by avidity. In the case of an immobilised multivalent ligand, clustering is achieved through the mobility of the receptor on the platelet surface as illustrated for the binding of platelet GPVI to collagen, as shown using single molecule super-resolution microscopy [40]. On the other hand, the clustering of CLEC-2 would require either a degree of prior clustering of podoplanin in the membrane or the formation of clusters as a result of binding and diffusion. The clustering of podoplanin could be achieved through binding of its cytosolic tail to the cytoskeletal via the ezrin, radixin and moesin (ERM) family of binding proteins [5] or in the case of a high level of expression by diffusion. The clustering and phosphorylation of CLEC-2 receptors to the podoplanin-rich areas would be reinforced by the binding of Syk to the CLEC-2 phosphorylated tail, thereby bringing in additional CLEC-2 receptors and further clustering of podoplanin. This has been demonstrated in lipid bilayers, with clustering of podoplanin by platelet CLEC-2 being dependent on Src and Syk tyrosine kinases [41]. The combined effect of avidity, diffusion and crosslinking by Syk counters the need for a high affinity interaction between podoplanin and CLEC-2 in mediating activation. In line with this, we have estimated the concentration of CLEC-2 in the membrane using to be below the reported affinity constant (*K*_D_) of 24 μM for binding to podoplanin [42] (see S1 Text). The net effect of affinity and avidity necessitates that competitive inhibitors would require a high affinity and a slow rate of dissociation for effective and sustained antagonism.

There are several factors that limit clustering of receptors under basal conditions and therefore unwanted activation. For platelet glycoprotein receptors, this includes their predominantly monomeric expression as illustrated for GPVI [32] and a low constitutive level of receptor phosphorylation as a result of a high tyrosine phosphatase activity at the membrane [6, 35, 43]. Receptors may also be prevented from dimerising by low affinity interactions with other membrane proteins, notably tetraspanins which have little biological activity on their own but have been shown to associate with platelet surface receptors [44]. It follows that a change in the distribution or activity of proteins that associate with or are located close to GPVI, such as tetraspanins and tyrosine phosphatases, has the potential to drive activation of glycoprotein receptors in the absence of ligand engagement.

There are a number of limitations to be considered in the models that have been developed in this study. In the ODE models, the single transmembrane receptors are considered to be monomeric, but in a cell there will be a mixture of dimers and higher order oligomers as discussed above for GPVI. The ODE and agent-based models are based on the presence of a single receptor for each ligands, but several ligands including collagen and fibrin bind to more than one receptor on the platelet surface. The modelling of a ligand with two different receptors is beyond the scope of this study.

**Fig 10:**
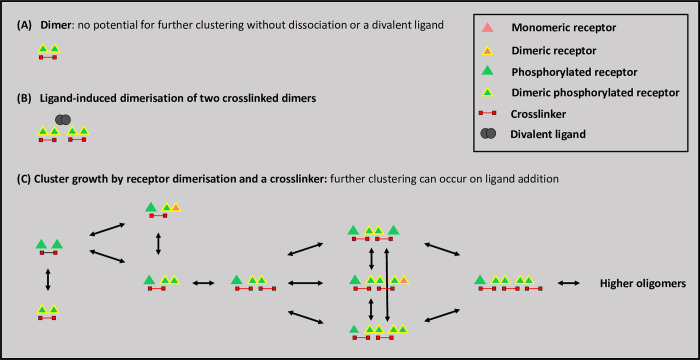
A model depicting how a combination of a weakly dimerising receptor and a crosslinking agent can drive cluster formation. The key is shown on the upper right: (A) illustrates crosslinking of two phosphorylated receptors in such a way that growth can only occur through dissociation or addition of a divalent ligands (B) ligand-induced dimerisation of two crosslinked dimers (C) cluster growth by a dimerising receptor and crosslinker. This can be further increased in the presence of a divalent ligand.

In summary, the models developed in this study highlight parameters that influence occupancy and clustering of weakly dimerising glycoprotein receptors, including ligand valency, receptor level and a cytosolic crosslinker. Predictions of the models have been tested through generation of novel divalent and tetravalent ligands, and combined use of inhibitors of ligand binding and Syk in functional studies. The results show that a tetravalent but not a divalent ligand induces powerful activation of human platelets demonstrating that 3 and possibly 4 ligands are required to induce activation. The absence of a bell-shaped dose response-relationship for the tetravalent ligand, in contrast to the ODE equlilbrium binding relationships, is like to reflect the existence of of pre-existing receptor dimers and other factors as discussed above. Fig 10 illustrates how receptor dimerisation and a cytosolic crosslinker combine to drive clustering, and how this can be increased in the presence of a divalent ligand. The synergy between low concentrations of a receptor blocker and a kinase inhibitor provides a strategy to overcome the effect of avidity and to lower the off-target effects of inhibitors.

There have been relatively few studies modelling activation of the platelet glycoprotein receptors that are the focus of this study. Martyanov and colleagues [45] have developed a stochastic multicompartmental computational model of CLEC-2 signalling which identifies clustering and signalosome formation as critical rate-limiting steps in platelet activation. The loss of downstream signalling accounts for the synergy between threshold concentrations of a GPVI-blocking nanobody and Syk inhibitor, even though Syk does not play a role in clustering of GPVI. The ability of Syk inhibition to block of CLEC-2 accounts provides an additional basis for the synergy and may explain why the inhibition does not recover at later times. Griffie *et al* [46] have modelled the clustering of receptors using ABM using a pre-set rule of “desire for clustering”. The application of this rule in the model leads to discrete clusters of receptors of uniform size (assuming a starting random distribution of the receptor). This study together with the models developed in thie manuscript illustrate how ABM can lead to a new understanding of the mechanisms of receptor clustering based on simple rules which can be tested in wet-lab experiments.

## Methods and materials

### Ethics statement

Healthy human volunteers for blood donation gave written consent under the Programme of Work ERN_11–0175 “The regulation of activation of platelets” ERN_11-0175P which was approved by the Science, Technology, Engineering and Mathematics Ethical Review Committee of the University of Birmingham.

### Modelling methods

#### Deterministic and stochastic modelling using ordinary differential equations (ODEs)

Systems of equations based on the law of mass action were developed to model the binding of monovalent, divalent and tetravalent soluble ligands, and monovalent membrane-bounds ligands to monomeric receptors. The numerical solutions for deterministic simulations were calculated using MATLAB’s in-built ODE solver ode15s, which was chosen for its advantage of being able to compute solutions for stiff differential equations (version 2019a). To compute stochastic simulations, the approach implemented was based on Gillespie’s algorithm [19]. Random number generators were used to introduce a degree of randomness for the time when a reaction would take place while considering the probability of each reaction occurring based on the rate constants, and the probability of the next reaction to occur was weighted by that of the previous reaction in addition to its respective rate constant. The outcome of x1000 iterations was combined and plotted against time.

Unless stated, the following parameters were used for simulations:

- Equilibrium dissociation constant (*K*_D_) = 1 μM
- Association rate constant (*k*_1_) = 1 μM^-1,^s^-1^
- Dissociation rate constant (*k*_-1_) = 1 s^-1^.

The scripts for developing ODE models can be found at: https://github.com/zeemaqsood/TAPAS_ESR9_Modelling_Project

### Agent-based modelling

ABM for monovalent, divalent and tetravalent ligands was performed using NetLogo [25] and used to study the interaction of such ligands with a monomeric receptor. The ABM models comprise of agents (which are termed ‘turtles’ in NetLogo) of multiple types, or breeds, and their interactions within the modelling environment based on defined rules. There are three main breeds: ligands, receptors and cross-linking proteins in the cytosol. Each breed possesses a unique set of attributes that can be changed and are responsible for conversion from one state to another as explained in the S1 Text and S6 Fig. Each run of 3,000 ticks was repeated 30 times, the results are shown as mean + s.d. The scripts for ABM model used can be found at: https://github.com/zeemaqsood/TAPAS_ESR9_Modelling_Project

### Experimental methods

#### Materials

Rhodocytin was purified from the venom of *Calloselasma rhodostoma* as described [47]. Atto-488 dye was obtained from Merck Life Science UK Limited (Dorset, UK). The Syk inhibitor PRT-060318 was purchased from Caltag Medsystems (Buckingham, UK). CRP was purchased from Cambcol Laboratories (Cambridge, UK). Other chemicals/reagents were obtained from Merck Life Science UK Limited or Polysciences (Pennsylvania, USA).

#### Antibodies and nanobodies

Nanobodies were raised against GPVI extracellular region through VIB Nanobody core (VIB Nanobody Service Facility, Brussels, Belgium). The most potent of these, Nb2, was expressed and purified as previously described [24]. Divalent (Nb2-2) and tetravalent (Nb2-4) versions of Nb2 were cloned by VIB Nanobody Core to contain a flexible (GGGGS)_3_ linker [48, 49] between each individual Nb2 domain, see S2A Fig for domain architecture. Expression and purification of Nb2-2 and Nb2-4 was as described for monovalent Nb2: *E*.*coli* WK6 cells were used to express the Nb construct which contained an N-terminal PelB leader sequence to secrete protein into the periplasmic space. The constructs also contained a C-terminal His-6 tag which was used for purification by nickel affinity chromatography. The mouse mAb AYP1 was raised against the C-type lectin-like domain of human CLEC-2 and Fab and F(ab)_2_ fragments generated as described [50].

#### Platelet preparation and light transmission aggregometry

Blood was obtained from healthy volunteers who had denied taking antiplatelet medication in the previous 10 days using 4% sodium citrate.

Washed human platelets were obtained by centrifugation of platelet-rich plasma in the presence of 0.2 μg/mL prostacyclin, resuspended in modified Tyrode’s (134 mM NaCl, 0.34 mM Na_2_HPO_4_, 2.9 mM KCl, 12 mM NaHCO_3_, 20 mM HEPES, 5 mM glucose, 1 mM MgCl_2_, pH 7.3) and allowed to rest for 30 min at room temperature. Washed platelets at 2x10^8^/mL were incubated with vehicle, PP2 (20 μM), PRT-060318 (10 μM) and Nb2 (100 nM) for 10 min, 37°C before stimulation with the stated concentration of CRP, collagen, Nb2, Nb2-2 and Nb2-4 in a Chronolog model 700 aggregometer or PAP-8E aggregometer while stirring at 1200 rpm, 37°C. The aggregation traces were monitored for up to 60 min. For the synergism studies, washed platelets at 2x10^8^/mL were incubated with vehicle, PRT-060318 (2.5 nM) and/or the divalent anti-CLEC-2 antibody, AYP1 F(ab)_2_ at 2.2 nM (individually or a combination of PRT-060318 and AYP1 F(ab)_2_) and/or the divalent anti-GPVI nanobody, Nb2-2 (2.2 nM) (individually or in combination of PRT-060318 and Nb2-2) for 10 min and then stimulated with rhodocytin (100nM) or CRP (1 μg/ml), respectively. Platelet aggregation was monitored using a Chronolog model 490 4+4 (Labmedics, Manchester, UK) for 10 min at 1200 rpm, 37°C.

#### Human CLEC-2-eGFP and GPVI-eGFP construct generation

cDNA for human CLEC1b (the gene encoding CLEC-2) with a linker coding for DRNLPPLAPL was synthesised (GeneArt, Invitrogen). The N-terminally eGFP tagged CLEC-2 construct was made by subcloning Clec1b cDNA in the pEGFP(A206K)-C1 expression vector for expression of CLEC-2-eGFP where the N-terminal eGFP has a A206K mutation to prevent dimerisation of the fluorescent protein. Generation of the GPVI-eGFP construct has been described previously [32].

#### Fluorescence correlation spectroscopy (FCS) and analysis

HEK293T cells were seeded in phenol red-free DMEM at a density of 3x10^4^ cells/25mm coverslip (Marienfeld, high precision, thickness No. 1.5H (0.170 mm ± 0.005 mm). The next day, cells were transiently transfected using polyethylenimine (PEI Max MW 40,000, Polysciences) (PEI:DNA ratio = 3:1, 3 μl:1 μg) where 100 ng CLEC-2-eGFP or 100 ng GPVI-eGFP + 100 ng FcRγ-chain DNA was used to achieve optimal receptor density. Cells were left growing a further 24 h at 37°C/5% CO_2_. FCS measurements were made using a Zeiss LSM-880 confocal microscope equipped with gallium arsenide phosphide photon detectors (GaAsP) (Carl Zeiss, Jena, Germany) and prior to each experiment the microscope was aligned and calibrated using Atto-488 dye as described previously [32]. On the day of the experiment, the FCS confocal volume was determined by measurement of the axial and lateral radii. For FCS experiments with ligands, rhodocytin (30 nM), AYP1 mAb (6.6 nM), CRP (10 μg/ml), Nb2-2 (10 nM) and Nb2-4 (10 nM) were made in phenol red-free DMEM and added to the cells and imaged immediately. For PRT samples, PRT-060318 (10 μM) was made in phenol red- free DMEM and added to cells for 45 min prior to ligand addition and imaging. DMSO only (0.05 and 0.1%) samples were imaged as a control. FCS data were analysed by photon counting histogram (PCH) analysis to determine molecular brightness using Zen 2012 (black edition) software (Carl Zeiss, Jena, Germany) as described previously [32].

#### Surface plasmon resonance

Surface plasmon resonance experiments were performed using a Biacore T200 instrument (Cytiva). GPVI (extracellular region) was immobilised directly on the CM5 chip using amine-coupling to the carboxylmethylated dextran-coated surface. Reference surfaces were blocked using 1M ethanolamine pH 8. All sensograms shown are double reference subtracted and at least two replicates were injected per cycle as well as experimental replicates of n = 3. Experiments were performed at 25°C with a flow rate of 30 μl/min in HBS-EP running buffer (0.01 M HEPES pH 7.4, 0.15 M NaCl, 3 mM EDTA, 0.005% v/v surfactant P20). Multi-cycle kinetic assays were used with at least 5 concentration points between 0.1x and 10x the *K*_D_. Each concentration of analyte (GPVI nanobody divalent/tetravalent) was run as follows, 120 sec injection, 900 sec dissociation, 30 sec regeneration with 10 mM glycine pH 1.5 followed by a 300 sec stabilisation period. Kinetic analysis was performed using the Biacore T200 Evaluation software using a global fitting to a 1:1 binding model.

### Statistical analysis

Results are shown as mean ± s.d. unless otherwise stated and the number of independent experiments is described in Figure legends. Dose response curves were fitted using dose-response fit in Origin (version 8.6, OriginLab, Northampton, MA). For FCS, data sets were first tested for normality using the Shapiro-Wilks test. FCS data were analysed by Kruskal-Wallis with Dunn’s *post-hoc* test using PRISM v9.2.0 (GraphPad, San Diego, CA). For the effect of inhibitors on aggregation, all treatment groups were first compared with each other using one-way ANOVA analysis, followed by multiple comparison tests. Significance was set at P ≤ 0.05.

## Supporting information

S1 Text Supplementary results, methods and references. (DOCX)

S1 Fig The interaction of a soluble monovalent ligand and a monomeric receptor. The graphical plots in (A) and (B) are based on Eq 1.5. (A) The effect of varying the ligand concentration on the time course of receptor occupancy. The ligand concentrations in (i), (ii) and (iii) were chosen to achieve 25, 50 and 75% receptor occupancy at equilibrium, with 95% equilibrium reached at 2.25, 1.50 and 0.75 sec, respectively. The times were derived using Matlab code shown below. (B) The effect of varying the receptor concentration on the time course of receptor occupancy for a ligand concentration of 1 μM and a receptor concentration of 0.1, 1 and 10 μM. The lines are superimposable as predicted by Eq 1.5. (C) The stochastic model of ligand-receptor interaction. The receptor numbers in (i), (ii) and (iii) are 10, 100 and 1,000 respectively. The bold line represents the mean and the hashed line the 5 and 95% values. The following parameters were used in all Figs: *K*_D_ = 1 μM, *k*_1_ = 1 μM^-1^s^-1^, *k*_-1_ = 1 s^-1^; they were chosen for illustration. The graphs were generated with the MATLAB code: https://github.com/zeemaqsood/TAPAS_ESR9_Modelling_Project.
(TIF)

S2 Fig Domain structure and further characterisation of nanobody ligands. (A) The domain structure of Nb2, Nb2-2 and Nb2-4 is shown along with the position of the (GGGGS)_3_ linker. (B) SDS-PAGE gel showing purified Nb2-2 and Nb2-4 protein, visualised using Coomassie stain.
(TIF)

S3 Fig Surface plasmon resonance data showing Nb2 divalent (Nb2-2) and tetravalent (Nb2-4) ligand binding to GPVI immobilised on a surface. Representative sensograms are shown. The binding affinity was determined by kinetic analysis, results are mean+s.e.m. of 3 experiments. The *K*_d_ for Nb2-2 and Nb2-4 are 0.10 + 0.01 nM and 0.20 + 0.10 nM, respectively. Data for the Nb2 monomer (*K*_d_ = 0.58 + 0.06 nM) is published in [24].
(TIF)

S4 Fig An agent-based model showing that the role of phosphorylation, receptor dimerisation and a moderate affinity crosslinker on receptor clustering. Agent-based modelling of the effect of phosphorylation, receptor dimerisation and the presence of a moderate affinity cytosolic crosslinker on receptor clustering. Unless stated, the parameter values and key are as described in Fig 4. (A) The effect of a moderate affinity crosslinker on formation of receptor dimers for a receptor that is unable to dimerise (monomeric receptor) (i) representative runs at steady-state, the number of moderate affinity crosslinkers is varied from 25–200 as shown in the upper right-hand corner (ii) number of receptors that are phosphorylated at steady-state (iii) number of receptor dimers, trimers, tetramers and higher order structures at steady-state. Results are shown as mean + s.d. of 30 simulations at 3,000 ticks. (B) The effect of a moderate affinity crosslinker on clustering of a receptor with a low starting level of dimerisation (10%) (i) the number of crosslinkers in the upper right-hand corner, for further details see (A). (C) The effect of receptor phosphorylation on clustering of receptors in the presence of a moderate affinity crosslinker (i) the rate of receptor phosphorylation per box is shown in ticks in the upper right-hand corner, for further details see (A). (D) The effect of receptor dimerisation on clustering of receptors in the presence of a moderate affinity crosslinker (i) the rate of receptor dimerisation is shown in ticks in the upper right-hand corner, for further details see (A). (E) The effect of a moderate affinity crosslinker on clustering of receptors in the presence of a high background of receptor dimerisation and phosphorylation. The rate of phosphorylation and degree of dimerisation are set to the highest values in (C) and (D), achieving ~80% and ~75%, respectively (i) The number of moderate affinity crosslinkers is shown in the upper right-hand corner, for further details see (A).
(TIF)

S5 Fig The Syk inhibitor PRT-060318 does not inhibit GPVI clustering by multivalent ligands. (i) Representative confocal microscopy images showing membrane localisation of GPVI-eGFP + FcRγ-chain resting and treated with collagen-related peptide (CRP, 10 μg/ml), divalent nanobody (Nb) Nb2-2 (10 nM), tetravalent Nb2-4 (10 nM), PRT-060318 (PRT) (10 μM) and PRT + multivalent ligands as labelled, in transfected HEK293T cells (field of view = 52 x 52 μm) (scale bar = 5 μm). (Aii) Box plots showing the effect of CRP (10 μg/ml), divalent Nb2-2 (10 nM), tetravalent Nb2-4 (10 nM), PRT (10 μM) and PRT + multivalent ligands on the molecular brightness (cpm s^-1^) of GPVI-eGFP. For all box plots, centre lines represent the median; box limits indicate the 25^th^ and 75^th^ percentiles and whiskers extend to minimum and maximum points. Significance was measured with Kruskal-Wallis with Dunn’s *post-hoc* where *P* ≤ 0.05. * = significance compared to GPVI alone (no ligand). FCS measurements were taken in 20–47 cells (n = 3).
(TIF)

S6 Fig State diagram of the agent-based clustering model. The Figure illustrates all possible states of all species comprising the ABM model and how these change based on inter as well as intra species interactions. There are six reactions occurring on three species: a divalent ligand (L^2^), receptor (R) and a crosslinker (S). A ligand-receptor complex formation reaction is coloured red, receptor phosphorylation (either basal or post ligand attachment to a receptor) is coloured blue, receptor-crosslinker complex formation is coloured green (either to an unbound or partially bound crosslinker), and receptor-receptor dimerisation reaction is coloured yellow.
(TIF)

S1 Table Protein expression levels in platelets. The expression levels of monomeric receptors and Syk. The levels are taken from quantitative proteomic studies in human [51] and mouse platelets [52].
(TIF)

S2 Table Turtle breeds and their attributes. All turtles are differentiated into three main breeds namely ligands, receptors and cytosolic cross-linkers. Each breed possesses a unique set of attributes that can be changed. The attributes are responsible for conversion of the state of each agent. The main attributes for each breed are listed in the Table below.
(TIF)

S1 Data Excel spreadsheet containing, in separate sheets, the underlying numerical data and statistical analysis for Fig panels 3A, 3B, 7A, 7B, 8A, 8B, S3 and S5. (XLSX)

S1 Video The effect of receptor number on recept imerizationion. The effect of increasing the receptor number on a weakly dimerising receptor. The number of receptors is shown in the upper right-hand corner. Receptor collisions and consequently the degree imerizationion increases with receptor density. For details see Fig 4A.
(MP4)

S2 Video The effect of a crosslinker imerizationion of a monomeric receptor. The effect of increasing the number of a high affinity crosslinker on a non-dimerising (monomeric) receptor at a low basal level of receptor phosphorylation (~10%). The number of crosslinkers is shown in the upper right-hand corner; the number of receptors is constant at 100 per box. The degree of phosphorylation increases with crosslinker density but as expected there are no clusters formed. For details see Fig 5A.
(MP4)

S3 Video The effect of a crosslinker on dimerisation of a weakly dimerising receptor. The effect of increasing the number of a high affinity crosslinker on a weakly dimerising receptor at a low basal level of receptor phosphorylation (~10%). The number of crosslinkers is shown in the upper right-hand corner; the number of receptors is constant at 100 per box. The degree of phosphorylation increases with crosslinker density but there is a marginal effect on receptor clustering. For details see Fig 5B.
(MP4)

S4 Video The effect of increasing the rate of phosphorylation of a weakly dimerising receptor in the presence of a crosslinker. The effect of increasing the on rate of phosphorylation of a weakly dimerising receptor in the presence of a high affinity crosslinker. The on rate of receptor phosphorylation is shown in the upper right-hand corner, and the number of receptors and crosslinkers is constant at 100 each per box. The degree of dimerisation and higher order clustering, as well as the overall receptor phosphorylation increases with an increase in the on rate of receptor phosphorylation. For details see Fig 5C.
(MP4)

S5 Video The effect of increasing the rate of receptor dimerisation in the presence of a crosslinker and low level of receptor phosphorylation. The effect of increasing the on rate of receptor dimerisation in the presence of a high affinity crosslinker and a low level of receptor phosphorylation (~10%). The on rate of receptor dimerisation is shown in the upper right-hand corner; the number of receptors and crosslinkers is constant at 100 each per box. The degree of dimerisation, higher order clustering and receptor phosphorylation increases with an increase in the on rate of receptor dimerisation. For details see Fig 5D.
(MP4)

S6 Video The time course of clustering of a weakly dimerising receptor in the presence of a divalent ligand and crosslinker. The time course of clustering of a divalent ligand in the presence of a moderate affinity crosslinker with a low starting level of dimerisation (10%). The tick intervals are shown in the upper right-hand corner. For details see Fig 6C.
(MP4)

## References

[1] Rayes J, Watson SP, Nieswandt B. Functional significance of the platelet immune receptors GPVI and CLEC-2. J Clin Invest. 2019, 129:12–23. doi: 10.1172/JCI122955 . 30601137 PMC6307936

[2] Kardeby C, Damaskinaki F, Sun Y, Watson SP. Is the endogenous ligand for PEAR1 a proteoglycan: clues from the sea. Platelets. 2021, 32:779–85. doi: 10.1080/09537104.2020.1863938 . 33356751

[3] Nieswandt B, Watson SP. Platelet-collagen interaction: is GPVI the central receptor? Blood. 2003, 102:449–61. doi: 10.1182/blood-2002-12-3882 . 12649139

[4] Arman M, Krauel K. Human platelet IgG Fc receptor FcγRIIA in immunity and thrombosis. J Thromb Haemost. 2015 Jun, 13:893–908. doi: 10.1111/jth.12905 . 25900780

[5] Martin EM, Zuidscherwoude M, Morán LA, Di Y, García A, Watson SP. The structure of CLEC-2: mechanism of dimerisation and higher order clustering. Platelets. 2021, 32:733–43. doi: 10.1080/09537104.2021.1906407 . 33819136

[6] Mazharian A, Wang YJ, Mori J, Bem D, Finney B, Heising S, et al. Mice lacking the ITIM-containing receptor G6b-B exhibit macrothrombocytopenia and aberrant platelet function. Science Signaling. 2012, 5:ra78. doi: 10.1126/scisignal.2002936 . 23112346 PMC4973664

[7] Mazharian A, Mori J, Wang YJ, Heising S, Neel BG, Watson SP, et al. Megakaryocyte-specific deletion of the protein-tyrosine phosphatases Shp1 and Shp2 causes abnormal megakaryocyte development, platelet production and function. Blood. 2013, 121: 4205–20. doi: 10.1182/blood-2012-08-449272 . 23509158 PMC3656453

[8] Zhang H, Cordoba SP, Dushek O, van der Merwe PA. Basic residues in the T-cell receptor zeta cytoplasmic domain mediate membrane association and modulate signaling. Proc Natl Acad Sci USA. 2011, 108:19323–8. doi: 10.1073/pnas.1108052108 . 22084078 PMC3228420

[9] Clark AJ. The mode of action of drugs on cells. Baltimore, MD: Williams and Wilkins Company; 1933.

[10] Monod J, Wyman J, Changeux J-P. On the nature of allosteric transitions: A plausible model. J Mol Biol. 1965;12:88–118. doi: 10.1016/s0022-2836(65)80285-6 . 14343300

[11] Kenakin TP. Principles: receptor theory in pharmacology. Trends Pharmacol Sci 2004; 25:186–92 doi: 10.1016/j.tips.2004.02.012 15063082

[12] Cooper JA, Qian H. A mechanism for SRC kinase-dependent signaling by noncatalytic receptors. Biochemistry. 2008, 47:5681–88. doi: 10.1021/bi8003044 . 18444664 PMC2614901

[13] Holcombe M, Adra S, Bicak M, Chin S, Coakley S, Graham AI, et al. Modelling complex biological systems using an agent-based approach. Integr Bio (Cam). 2012, 4:53–64. doi: 10.1039/c1ib00042j . 22052476

[14] Bonabeau E. Agent-based modeling: Methods and techniques for simulating human systems. Proc. Natl. Acad. Sci. 2002, 99: 7280–7287. doi: 10.1073/pnas.082080899 12011407 PMC128598

[15] An G. A model of TLR4 signaling and tolerance using a qualitative, particle-event-based method: introduction of spatially configured stochastic reaction chambers (SCSRC). Math Biosci. 2009, 217:43–52. doi: 10.1016/j.mbs.2008.10.001 . 18950646

[16] Das AA, Ajayakumar Darsana T, Jacob E. Agent-based re-engineering of ErbB signaling: a modeling pipeline for integrative systems biology. Bioinformatics. 2017, 33:726–32. doi: 10.1093/bioinformatics/btw709 . 27998938

[17] Mackay D. The mathematics of drug-receptor interactions. J. Pharm. Pharmacol. 1966 Apr, 18:201–22. doi: 10.1111/j.2042-7158.1966.tb07854.x . 4381245

[18] Lauffenburger DA, Linderman JJ. Receptors: models for binding, trafficking, and signaling. Oxford University Press; 1996. Page 20.

[19] Gillespie DT. A general method for numerically simulating the stochastic time evolution of coupled chemical reactions. J Comput Phys. 1976, 22:403–34.

[20] Hermans J, Lentz B. Equilibria and Kinetics of Biological Macromolecules. John Wiley & Sons, Inc; 2013. p. 226.

[21] White C, Bridge LJ. Ligand binding dynamics for pre-dimerised G protein-coupled receptor homodimers: Linear models and analytical solutions. Bull Math Biol. 2019, 81:3542–74. doi: 10.1007/s11538-017-0387-x . 29349610 PMC6722261

[22] White C, Rottschäfer V, Bridge LJ. Insights into the dynamics of ligand-induced dimerisation via mathematical modelling and analysis. J. Theor. Biol. 2022, 538:110996. doi: 10.1016/j.jtbi.2021.110996 . 35085533

[23] Montague SJ, Patel P, Martin EM, Slater A, Quintanilla LG, Perrella G, et al. Platelet activation by charged ligands and nanoparticles: platelet glycoprotein receptors as pattern recognition receptors. Platelets. 2021, 32:1018–30. doi: 10.1080/09537104.2021.1945571 . 34266346

[24] Slater A, Di Y, Clark J, Jooss NJ, Martin EM, Alenazy F, et al. Structural characterisation of a novel GPVI nanobody-complex reveals a biologically active domain-swapped GPVI dimer. Blood. 2021, 137:3443–53. doi: 10.1182/blood.2020009440 . 33512486

[25] Tisue S, Wilensky U. NetLogo: Design and implementation of a multi-agent modeling environment. Proceedings of the Agent 2004 Conference on Social Dynamics: Interaction, Reflexivity and Emergence; 2004 Oct 7–9; Chicago, IL.

[26] Pawson T Specificity in signal transduction: from phosphotyrosine-SH2 domain interactions to complex cellular systems. Cell, 2004; 116:191–203. doi: 10.1016/s0092-8674(03)01077-8 14744431

[27] Meseth U, Wohland T, Rigler R and Vogel H. Resolution of fluorescence correlation measurements. Biophys J. 1999, 76:1619–31. doi: 10.1016/S0006-3495(99)77321-2 . 10049342 PMC1300138

[28] Chen Y, Müller JD, So PT, Gratton E. The photon counting histogram in fluorescence fluctuation spectroscopy. Biophys J. 1999, 77:553–67. doi: 10.1016/S0006-3495(99)76912-2 . 10388780 PMC1300352

[29] Severin S, Pollitt AY, Navarro-Nunez L, Nash CA, Mourao-Sa D, Eble JA, et al. Syk-dependent phosphorylation of CLEC-2: a novel mechanism of hem-immunoreceptor tyrosine-based activation motif signaling. J Biol Chem. 2011, 286:4107–16. doi: 10.1074/jbc.M110.167502 . 21098033 PMC3039337

[30] Nagy M, Perrella G, Dalby A, Becerra MF, Garcia Quintanilla L, Pike JA, et al. Flow studies on human GPVI-deficient blood under coagulating and non-coagulating conditions. Blood Adv. 2020, 4:2953–61. doi: 10.1182/bloodadvances.2020001761 . 32603422 PMC7362345

[31] Snell DC, Schulte V, Jarvis GE, Arase K, Sakurai D, Saito T, et al. Differential effect of reduced glycoprotein VI levels on activation of murine platelets by glycoprotein VI ligands. Biochem. J. 2002, 368:293–300. doi: 10.1042/BJ20020335 . 12117414 PMC1222953

[32] Clark JC, Neagoe RAI, Zuidscherwoude M, Kavanagh DM, Slater A, Martin EM, et al. Evidence that GPVI is expressed as a mixture of monomers and dimers, and that the D2 domain is not essential for GPVI activation. Thromb. Haemost. 2021, 121:1435–47. doi: 10.1055/a-1401-5014 . 33638140

[33] Tomlinson MG, Calaminus SD, Berlanga O, Auger JM, Bori-Sanz T. Collagen promotes sustained glycoprotein VI signaling in platelets and cell lines. J. Thromb. Haemost. 2007, 5:2274–83. doi: 10.1111/j.1538-7836.2007.02746.x . 17764536

[34] Watson CN, Kerrigan S, Cox D, Henderson I, Watson SP, Arman M. Human platelet activation by Escherichia coli: roles for FcγRIIA and integrin αIIbβ3. Platelets. 2016, 27: 535–40. doi: 10.3109/09537104.2016.1148129 . 27025455 PMC5000871

[35] Blake R, Walker T, Watson SP. Activation of human platelets by peroxovanadate is associated with tyrosine phosphorylation of phospholipase C γ and formation of inositol phosphates. Biochem. J. 1993, 290:471–5. doi: 10.1042/bj2900471 . 8452536 PMC1132297

[36] Suzuki-Inoue K, Fuller GL, Garcia A, Eble JA, Pohlmann S, Inoue O, et al. A novel Syk-dependent mechanism of platelet activation by the C-type lectin receptor CLEC-2. Blood. 2006, 107:542–9. doi: 10.1182/blood-2005-05-1994 . 16174766

[37] Watson SP, Reep B, McConnell RT, Lapetina EG. Collagen stimulates inositol trisphosphate formation in indomethacin–treated human platelets. Biochem. J. 1985, 226:831–37. doi: 10.1042/bj2260831 . 3872656 PMC1144783

[38] Smith C, Montague S, Kardeby C, Di Y, Lowe G, Lester W, et al. Anti-platelet drugs block platelet activation by vaccine-induced immune thrombocytopenia and thrombosis patient serum. Blood. 2021, 138:2733–40. doi: 10.1182/blood.2021012277 . 34375398 PMC8697531

[39] Arman M, Krauel K, Tilley DO, Weber C, Cox D, Greinacher A, et al. Amplification of bacteria-induced platelet activation is triggered by FcγRIIA, integrin αIIbβ3 and platelet factor 4. Blood. 2014, 123:3166–74. doi: 10.1182/blood-2013-11-540526 . 24642751 PMC4023422

[40] Pallini C, Pike JA, O’Shea C, Andrews RK, Gardiner EE, Watson SP, et al. Immobilised collagen prevents shedding and induces sustained GPVI clustering and signalling in platelets. Platelets. 2021, 32:59–73. doi: 10.1080/09537104.2020.1849607 . 33455536

[41] Pollitt AY, Poulter NS, Gitz E, Navarro-Nunez L, Wang YJ, Hughes CE, et al. Syk and Src family kinases regulate C-type lectin receptor 2 (CLEC-2)-mediated clustering of podoplanin and platelet adhesion to lymphatic endothelial cells. J Biol Chem. 2014, 289:35695–710. doi: 10.1074/jbc.M114.584284 . 25368330 PMC4276840

[42] Christou CM, Pearce AC, Watson AA, Mistry AR, Pollitt AY, Fenton-May AE, et al. Renal cells activate the platelet receptor CLEC-2 through podoplanin. Biochem J. 2008, 411:133–40. doi: 10.1042/BJ20071216 . 18215137 PMC2749330

[43] Senis Y, Tomlinson MG, Ellison S, Mazharian A, Lim J, Kornerup KN, et al. The tyrosine phosphatase CD148 is an essential positive regulator of platelet activation and thrombosis. Blood. 2009, 113: 4942–54. doi: 10.1182/blood-2008-08-174318 . 19246339 PMC2686144

[44] Haining E, Yang J and Tomlinson M. Tetraspanin microdomains: fine-tuning platelet function. Biochem Soc Trans. 2011, 39:518–23. doi: 10.1042/BST0390518 . 21428931

[45] Martyanov AA, Balabin FA, Dunster JL, Panteleev MA, Gibbins JM and Sveshnikova AN (2020) Control of platelet CLEC-2-mediated activation by receptor clustering and tyrosine kinase signalling. Biophys J. 118, 2641–2655. doi: 10.1016/j.bpj.2020.04.023 32396849 PMC7264845

[46] Griffie J, Peters R, Owen DM (2020) An agent-based model of molecular aggregation at the cell membrane. PLOS ONE 15: e0226825. doi: 10.1371/journal.pone.0226825 32032349 PMC7006917

[47] Eble JA, Beermann B, Hinz HJ, Schmidt-Hederich A. Alpha 2beta 1 integrin is not recognized by rhodocytin but is the specific, high affinity target of rhodocetin, an RGD-independent disintegrin and potent inhibitor of cell adhesion to collagen. J Biol Chem. 2001, 276:12274–84. doi: 10.1074/jbc.M009338200 . 11121411

[48] Klein JS, Jiang S, Galimidi RP, Keeffee J, Bjorkman PJ. Design and characterisation of structured protein linkers with differing flexibilities. Protein Eng Des Sel. 2014, 27:325–30. doi: 10.1093/protein/gzu043 . 25301959 PMC4191447

[49] Sadeghnezhad G, Romao E, Bernedo-Navarro R, Massa S, Khajeh K, Muyldermans S, et al. Identification of New DR5 agnosistic nanobodies and generation of multivalent nanobody constructs for cancer treatment. Int J Mol Sci. 2019, 20:4818. doi: 10.3390/ijms20194818 . 31569768 PMC6801735

[50] Gitz E, Pollitt AY, Gitz-Francois JJ, Alshehri O, Mori J, Montague S, et al. CLEC-2 expression is maintained on activated platelets and on platelet microparticles. Blood. 2014, 124:2262–70. doi: 10.1182/blood-2014-05-572818 . 25150298 PMC4183985

[51] Burkhart JM, Vaudel M, Gambaryan S, Radau S, Walter U, Martens L, et al. The first comprehensive and quantitative analysis of human platelet protein composition allows the comparative analysis of structural and functional pathways. Blood. 2012;120:e73–82. doi: 10.1182/blood-2012-04-416594 . 22869793

[52] Zeiler M, Moser M, Mann M. Copy number analysis of the murine platelet proteome spanning the complete abundance range. Mol Cell Proteom. 2014;13:3435–45. doi: 10.1074/mcp.M114.038513 . 25205226 PMC4256495

