# Supplementary material for: Experimental validation of computerised models of clustering of platelet glycoprotein receptors that signal via tandem SH2 domain proteins
Source: PLoS Comput Biol. 2022 Nov 28;18(11):e1010708. doi: 10.1371/journal.pcbi.1010708 (PMC9731471; doi:10.1371/journal.pcbi.1010708)
Supplement: S1 Text — (DOCX) [file pcbi.1010708.s001.docx]

**Experimental validation of computerised models of clustering of platelet glycoprotein receptors that signal via tandem SH2 domain proteins**

Zahra Maqsood^1,2,3¶^, Joanne C. Clark^1,4¶^, Eleyna M. Martin^1*¶^, Yam Fung Hilaire Cheung^1,5,6^, Luis A. Morán^1^, Sean E.T. Watson^1^, Jeremy A. Pike^1,4^, Ying Di^1^, Natalie S. Poulter^1,4^, Alexandre Slater^1^, Bodo M. H. Lange^2^, Bernhard Nieswandt^3^, Johannes A. Eble^7^, Mike G. Tomlinson^4,8^, Dylan M. Owen^4,9^, David Stegner^3^, Lloyd J. Bridge^10^, Christoph Wierling^2^ and Steve P. Watson^1,4,6^

1. Institute of Cardiovascular Sciences, IBR Building, College of Medical and Dental Sciences, University of Birmingham, Birmingham UK.
2. Alacris Theranostics, GmbH, Max-Planck-Straße 3, 12489 Berlin, Germany.
3. Rudolf Virchow Center for Integrative and Translation Bioimaging, University of Würzburg and Institute of Experimental Biomedicine I, University Hospital of Würzburg, Würzburg, Germany.
4. Centre of Membrane Proteins and Receptors (COMPARE), Universities of Birmingham and Nottingham, The Midlands, UK.
5. Leibniz-Institut für Analytische Wissenschaften – ISAS - e. V., Dortmund, Germany.
6. School of Biochemistry, Cardiovascular Research Institute Maastricht (CARIM), Maastricht University, Maastricht, The Netherlands.
7. Institute of Physiological Chemistry and Pathobiochemistry, University of Münster, Münster, Germany.
8. Department of Biosciences, University of Birmingham, Birmingham, UK.
9. Institute of Immunology and Immunotherapy, IBR Building, College of Medical and Dental Sciences, University of Birmingham, Birmingham, UK.
10. Faculty of Environment & Technology, Department of Computer Science and Creative Technologies, University of the West England, Frenchay Campus, Bristol, UK.

**^¶^**These authors contributed equally to the study

**Supplementary information**

**Results**

**ODE modelling**

**ODE modelling of the time course of binding of a monovalent ligand and a monomeric receptor**

S1 Fig illustrates the time course of interaction of a monovalent soluble ligand with a monomeric receptor for ligand concentrations that give 25, 50 and 75% receptor occupancy at equilibrium. The time to 95% of equilibrium decreases as the concentration of ligand increases (as shown by the dashed lines, S1A Fig), whereas the time to equilibrium does not change with the receptor concentration (S1B Fig). The stochastic nature of the interaction was modelled using Gillespie’s algorithm [1] as illustrated in S1C Fig.

**ODE modelling of the interaction of a tetravalent ligand and a monomeric receptor**

The binding of a ligand with four epitopes to a monomeric receptor will generate four different ligand-receptor species, and involves four equilibria defined by the order of attachment from unsaturated ligand (Eq 3.1) to fully saturated ligand (3.4):

|  | $\text{L}^{\text{4}}\text{ + R}\begin{matrix} k_{1} \\ \rightleftharpoons\\ k_{-1} \end{matrix}\text{L}^{\text{4}}\text{R}\boldsymbol{,}$ | (3.1) |
| --- | --- | --- |
|  | $\text{L}^{\text{4}}\text{R + R}\begin{matrix} k_{2}=\text{α}_{1}\cdot k_{1} \\ \text{ }\text{⇌}\text{ } \\ k_{-2}=\text{α}_{-1}\cdot k_{-1} \end{matrix}\text{L}^{\text{4}}\text{RR}\boldsymbol{,}$ | (3.2) |
|  | $\text{L}^{\text{4}}\text{RR + R}\begin{matrix} k_{3}=\text{α}_{2}\cdot k_{1} \\ \rightleftharpoons\\ k_{-3}=\text{α}_{-2}\cdot k_{-1} \end{matrix}\text{L}^{\text{4}}\text{RRR}\boldsymbol{,}$ | (3.3) |
|  | $\text{L}^{\text{4}}\text{RRR + R}\begin{matrix} k_{4}=\text{α}_{3}\cdot k_{1} \\ \rightleftharpoons\\ k_{-4}=\text{α}_{-3}\cdot k_{-1} \end{matrix}\text{L}^{\text{4}}\text{RRRR}\boldsymbol{.}$ | (3.4) |

The *K*_D_ of each reaction is defined as:

|  | $K_{\text{D}n}= \begin{matrix} \underline{k_{-n}} \\ k_{n} \end{matrix} = \begin{matrix} \underline{\text{ }\text{[L}^{4}\text{R}\text{(n-1)}\text{ ]∙ [R]}} \\ [\text{L}^{4}\text{(}n\text{R)]} \end{matrix}\boldsymbol{.}$ | (3.5) |
| --- | --- | --- |

**Estimating the concentration of a membrane-associated protein**

We have used two methods to estimate the concentration of a protein in a 2-dimensional space using CLEC-2 as a representative. This has been undertaken to investigate whether the concentration of CLEC-2 is in the range of its affinity constant for its endogenous ligand, the transmembrane protein podoplanin. We have focussed on CLEC-2 rather than podoplanin as the density of podoplanin in the membrane is not known and varies between cells. In the first method, we have estimated a length scale for the third dimension in order to generate a volume, and in the second method we have calculated the nearest neighbour assuming a uniform distribution in the membrane and extrapolated this to a third dimension. Both methods estimate the concentration of CLEC-2 to be below the reported *K_D_* values for binding of human monovalent and divalent podoplanin for monomeric CLEC-2 suggesting that avidity is a factor. This can be achieved by the clustering of proteins in the membrane either through a combination of diffusion and chance collisions or by self-association.

**Method 1: Estimating the volume**

This method is based on the estimation of the volume by considering the size of the objects under consideration and the location of their binding region. The height of CLEC-2 and podoplanin were estimated using the extracellular lengths of the glycoproteins CD45 and CD148. The extracellular domains of CD45 and CD148 are made up of 551 and 847 amino acid residues, and their sizes are estimated to be approximately 40 and 55 nm, respectively [2]. Therefore, the average extracellular size of each amino acid residue is about 0.07 nm, which is less than the average length of an amino acid molecule of 0.39 nm due to the compact secondary and tertiary structure. The extracellular domains of CLEC-2 and podoplanin consist of 172 and 108 amino acid residues, respectively, estimating the height of CLEC-2 and podoplanin above the membrane of 12 nm and 7.5 nm, respectively. As a separate approach, the height of CLEC-2 was estimated using the Alphafold prediction for full length CLEC-2 [3,4]. This gives a maximal height of 9.63 nm reflecting the folded nature of the protein. The binding sites of the two proteins are towards the upper end and so this volume will be much lower. For calculation purposes, we used an arbitrary distance of 2 nm, while the calculations above show that this could be much larger. Using Avogadro’s constant, this gives a concentration for 3,000 copies of CLEC-2 in a volume of 9.72 x 10^-14^ litres of approximately 50 nM.

**Method 2: Nearest neighbour distance**

This method is based on calculating the distance between evenly spaced ligands in the membrane and extrapolating this to a volume [5]. We have estimated the Nearest Neighbour Distance in 2-dimensions and from this we calculated the concentration in 3-dimensions.

The analytical expression for the expected nearest-neighbor distance in 2D (D_2D_) is given by

$D_{2D}=\frac{1}{2\sqrt{\rho_{2D}}}$ **Eq. S-1**

Where $\rho_{2D}$ is the molecule density in 2D (points per square micron)

For the 3D case we have $D_{3D}=\frac{1}{2\sqrt[3]{\rho_{3D}}}$ **Eq. S-2**

We set these equal to each other and we get $\rho_{3D}$ = $\rho_{2D}$^3/2^

We have 3000 molecules of CLEC-2 in 25 μm^2^ = a concentration of 120 molecules μm^2^.

Extrapolating to 3D (Eq. S-2) gives a concentration of 1315 molecules / μm^3^

To convert to Molar (M) concentration = 1315 x 10^15^ / N_A_ M

Where: N_A_ = Avogadro constant = 6.0 x 10^23^

1 x 10^15^ μm^3^ = 1 litre

= 219 x 10^-8^ M (2.2 μM).

**Methods**

**Agent-based model**

The state diagram in S6 Fig. summarises the flow of state changes followed by all three breeds of agents, and which themes govern each state change. The model is based on *themes*, which represent reaction types that can occur. The themes are applicable to each agent-based on:

1. state of the agent and
2. the breed class it belongs to.

A connection between two turtles (a turtle refers to single species or agent) is established and exploited on the basis of *links*. Each possible reaction within the system has a unique switch to turn the reaction on or off, and unique constants for forward and backward reactions. In general, three types of reactions are considered in the system:

1. Association ($A + B \underset{\to}{\text{k}_{\text{1}}} C$): wherein A reacts with B to form C at a given rate *k_1_*. Sometimes, C could be a final level product and if this happens to be the case, it may not take part in any more reactions to form any other product, i.e. a set of C’s may not be part of any further association reaction.
2. Dissociation ($C \underset{\to}{{-k}_{\text{1}}} A+B$): wherein C dissociates to form A and B at a given rate *-k*_1_.
3. Transformation ($D \underset{\to}{k_{2}} D^{*}; D^{*} \underset{\to}{{-k}_{2}} D$): wherein D transforms into its higher state and is able to revert back, governed by its unique set of rates constants *k*_2_ and -*k*_2_, respectively.

The list of themes, or reactions, included in the model is as follows:

1. Basal phosphorylation (and dephosphorylation) of receptors
2. Receptor dimerisation
3. Ligand dimerisation
4. Ligand-receptor complex formation
5. First attachment of cross-linker
6. Second attachment of cross-linker

Each theme can either be switched on or off, so that the effect of a subset of all themes can be investigated in isolation from other theme(s). The probability for each theme, or type of reaction, to occur either in the forward or, if applicable, backward reaction, is dependent on the probability value set by the user in the interface.

***Setup***

The setup function has the following sub routines:

- **setup-globals:** Initializes and sets all global variables.
- **setup-species:** Calls three further sub-routines which individually create three types of species namely L (ligands), R (receptors), and S (cross-linkers). The total number of units created for each species is based on user-defined values from the interface.
- **setup-dense-region:** Creates patches of dense regions in the world. The user can vary absence of presence of such regions, the density, size per region and the co-efficient of diffusion through the interface.

***go***

This function is responsible for calling subroutines associated with logic pertaining to reactions, updating reporters, exit strategy and displacement of agents:

- **exit logic**: Stops a run if either the number of ticks reaches the threshold specified by the user, or when the system reaches a steady-state.
- **move**: Defines the speed, direction of movement of all agents based on the property of patches they are found at. This sub-routine also defines the slowing down of clusters, inversely proportional to the mass of the clusters.

This function is also responsible for calling subroutines associated with reporting metrics and transformation, association or dissociation of agents as per reactions they partake in, depending on which themes are turned on/off and the probabilities set for each theme to go forward or backward, from within the interface.

Steady states were determined through calculation of the coefficient of variance for all species at the end of each iteration of the logic (or tick) and the state of the system was declared at equilibrium when this coefficient of variance was below a certain pre-defined threshold value.

**References**

1. Gillespie DT. A general method for numerically simulating the stochastic time evolution of coupled chemical reactions. J Comput Phys. 1976;22:403-34.
2. van der Merwe PA, Davis SJ, Shaw AS, Dustin ML. Cytoskeletal polarisation and redistribution of cell-surface molecules during T cell antigen recognition. Semin Immunol. 2000;12:5-21. PMID: 10723794.
3. Jumper J, Evans R, Pritzel A, Green T, Figurnov M, Ronneberger O, et al. Highly accurate protein structure prediction with AlphaFold. Nature. 2021; 596: 583-9. PMID: 34265844.
4. Varadi M, Anyango S, Deshpande M, Nair S, Natassi C, Yordanova G, et al. AlphaFold protein structure database: massively expanding the structural coverage of protein-sequence space with high-accuracy models. Nucleic Acids Res. 2022;50:D439-44. PMID: 34791371.
5. Clark PJ and Evans FC. Distance to nearest neighbour as a measure of spatial relationships in populations. Ecology. 1954;35:445-53.
