# Supplementary figures and images for: Experimental validation of computerised models of clustering of platelet glycoprotein receptors that signal via tandem SH2 domain proteins
Source: PLoS Comput Biol. 2022 Nov 28;18(11):e1010708. doi: 10.1371/journal.pcbi.1010708 (PMC9731471; doi:10.1371/journal.pcbi.1010708)

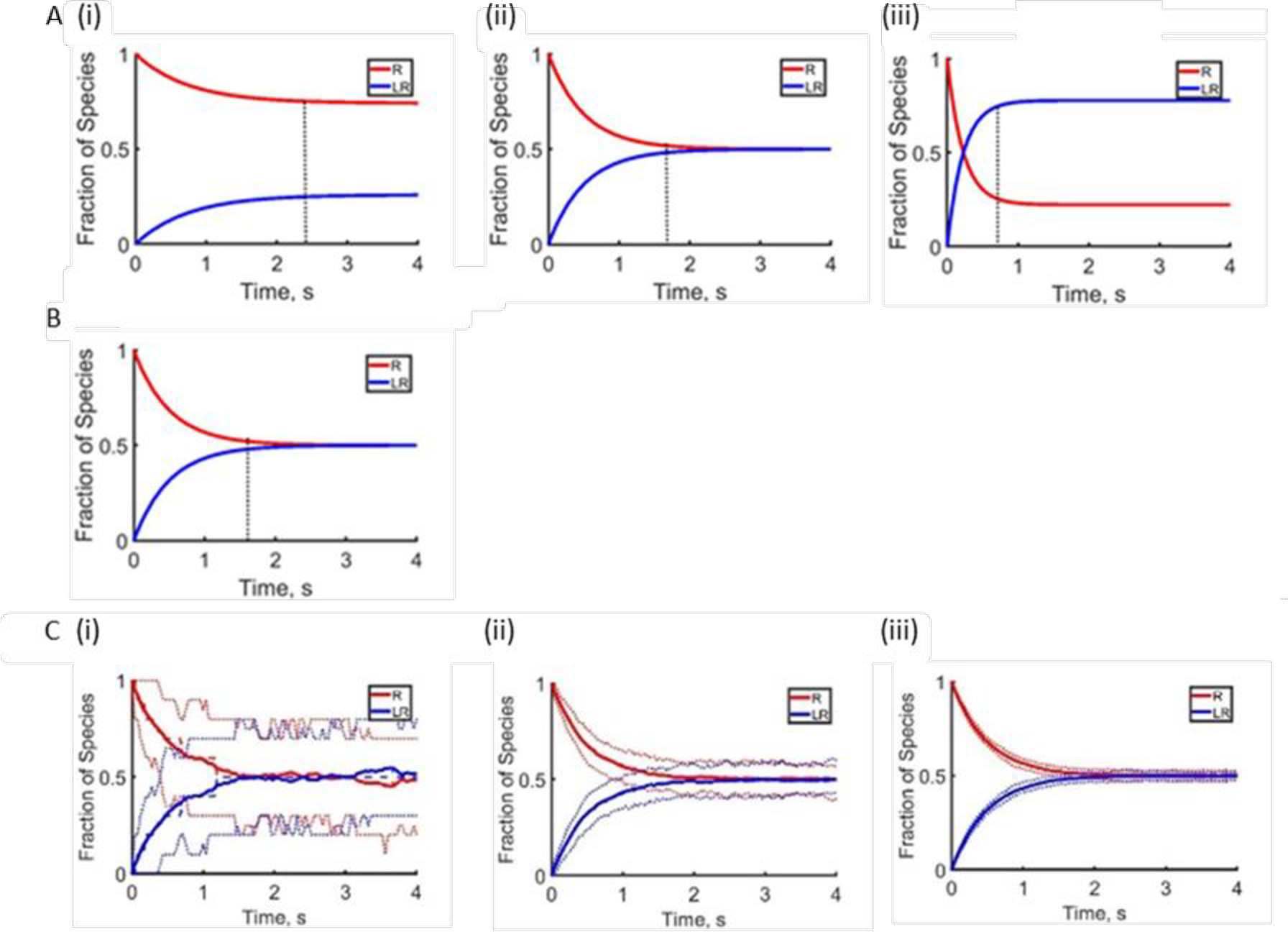

Supplement: S1 Fig — The graphical plots in (A) and (B) are based on Eq 1.5. (A) The effect of varying the ligand concentration on the time course of receptor occupancy. The ligand concentrations in (i), (ii) and (iii) were chosen to achieve 25, 50 and 75% receptor occupancy at equilibrium, with 95% equilibrium reached at 2.25, 1.50 and 0.75 sec, respectively. The times were derived using Matlab code shown below. (B) The effect of varying the receptor concentration on the time course of receptor occupancy for a ligand concentration of 1 μM and a receptor concentration of 0.1, 1 and 10 μM. The lines are superimposable as predicted by Eq 1.5. (C) The stochastic model of ligand-receptor interaction. The receptor numbers in (i), (ii) and (iii) are 10, 100 and 1,000 respectively. The bold line represents the mean and the hashed line the 5 and 95% values. The following parameters were used in all Figs: KD = 1 μM, k1 = 1 μM-1s-1, k-1 = 1 s-1; they were chosen for illustration. The graphs were generated with the MATLAB code: https://github.com/zeemaqsood/TAPAS_ESR9_Modelling_Project. (TIF) [file pcbi.1010708.s002.tif]

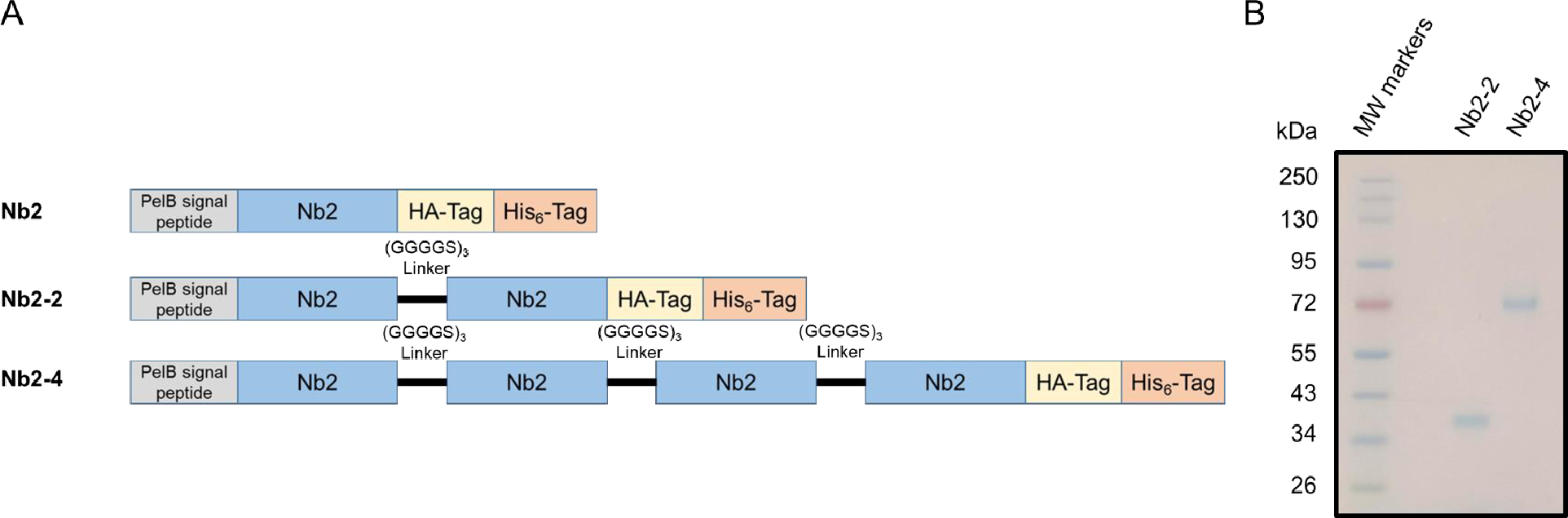

Supplement: S2 Fig — (A) The domain structure of Nb2, Nb2-2 and Nb2-4 is shown along with the position of the (GGGGS)3 linker. (B) SDS-PAGE gel showing purified Nb2-2 and Nb2-4 protein, visualised using Coomassie stain. (TIF) [file pcbi.1010708.s003.tif]

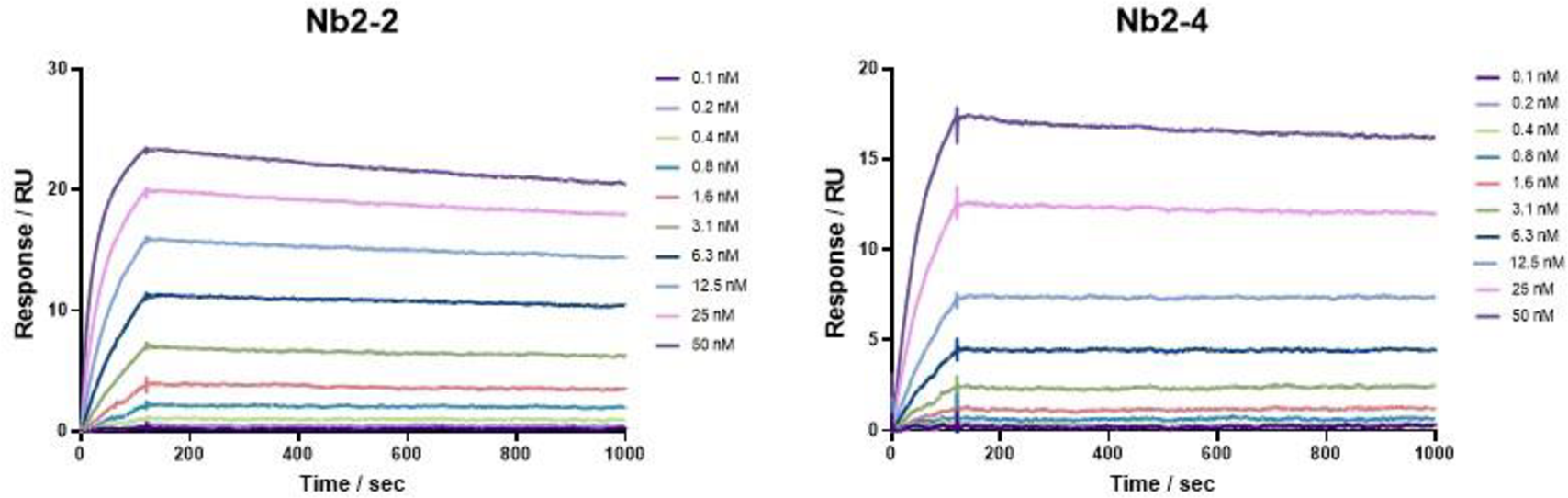

Supplement: S3 Fig — Representative sensograms are shown. The binding affinity was determined by kinetic analysis, results are mean+s.e.m. of 3 experiments. The Kd for Nb2-2 and Nb2-4 are 0.10 + 0.01 nM and 0.20 + 0.10 nM, respectively. Data for the Nb2 monomer (Kd = 0.58 + 0.06 nM) is published in [24]. (TIF) [file pcbi.1010708.s004.tif]

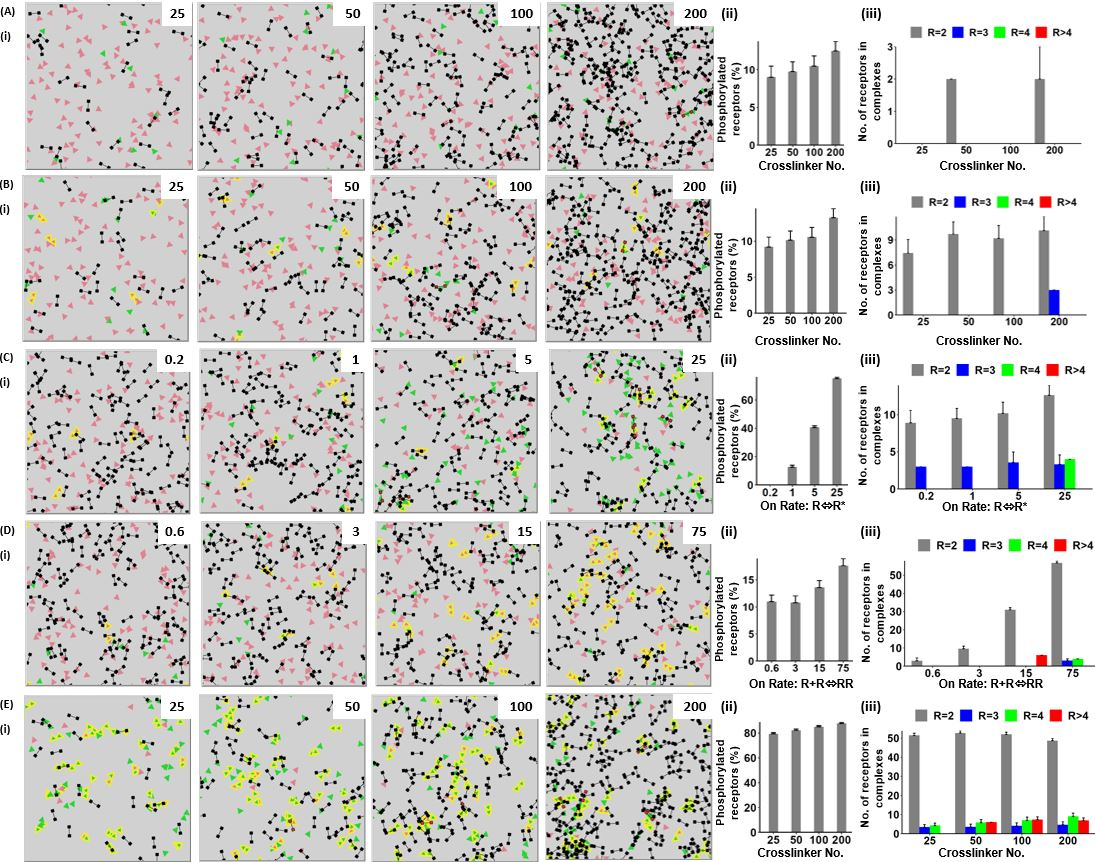

Supplement: S4 Fig — Agent-based modelling of the effect of phosphorylation, receptor dimerisation and the presence of a moderate affinity cytosolic crosslinker on receptor clustering. Unless stated, the parameter values and key are as described in Fig 4. (A) The effect of a moderate affinity crosslinker on formation of receptor dimers for a receptor that is unable to dimerise (monomeric receptor) (i) representative runs at steady-state, the number of moderate affinity crosslinkers is varied from 25–200 as shown in the upper right-hand corner (ii) number of receptors that are phosphorylated at steady-state (iii) number of receptor dimers, trimers, tetramers and higher order structures at steady-state. Results are shown as mean + s.d. of 30 simulations at 3,000 ticks. (B) The effect of a moderate affinity crosslinker on clustering of a receptor with a low starting level of dimerisation (10%) (i) the number of crosslinkers in the upper right-hand corner, for further details see (A). (C) The effect of receptor phosphorylation on clustering of receptors in the presence of a moderate affinity crosslinker (i) the rate of receptor phosphorylation per box is shown in ticks in the upper right-hand corner, for further details see (A). (D) The effect of receptor dimerisation on clustering of receptors in the presence of a moderate affinity crosslinker (i) the rate of receptor dimerisation is shown in ticks in the upper right-hand corner, for further details see (A). (E) The effect of a moderate affinity crosslinker on clustering of receptors in the presence of a high background of receptor dimerisation and phosphorylation. The rate of phosphorylation and degree of dimerisation are set to the highest values in (C) and (D), achieving ~80% and ~75%, respectively (i) The number of moderate affinity crosslinkers is shown in the upper right-hand corner, for further details see (A). (TIF) [file pcbi.1010708.s005.tif]

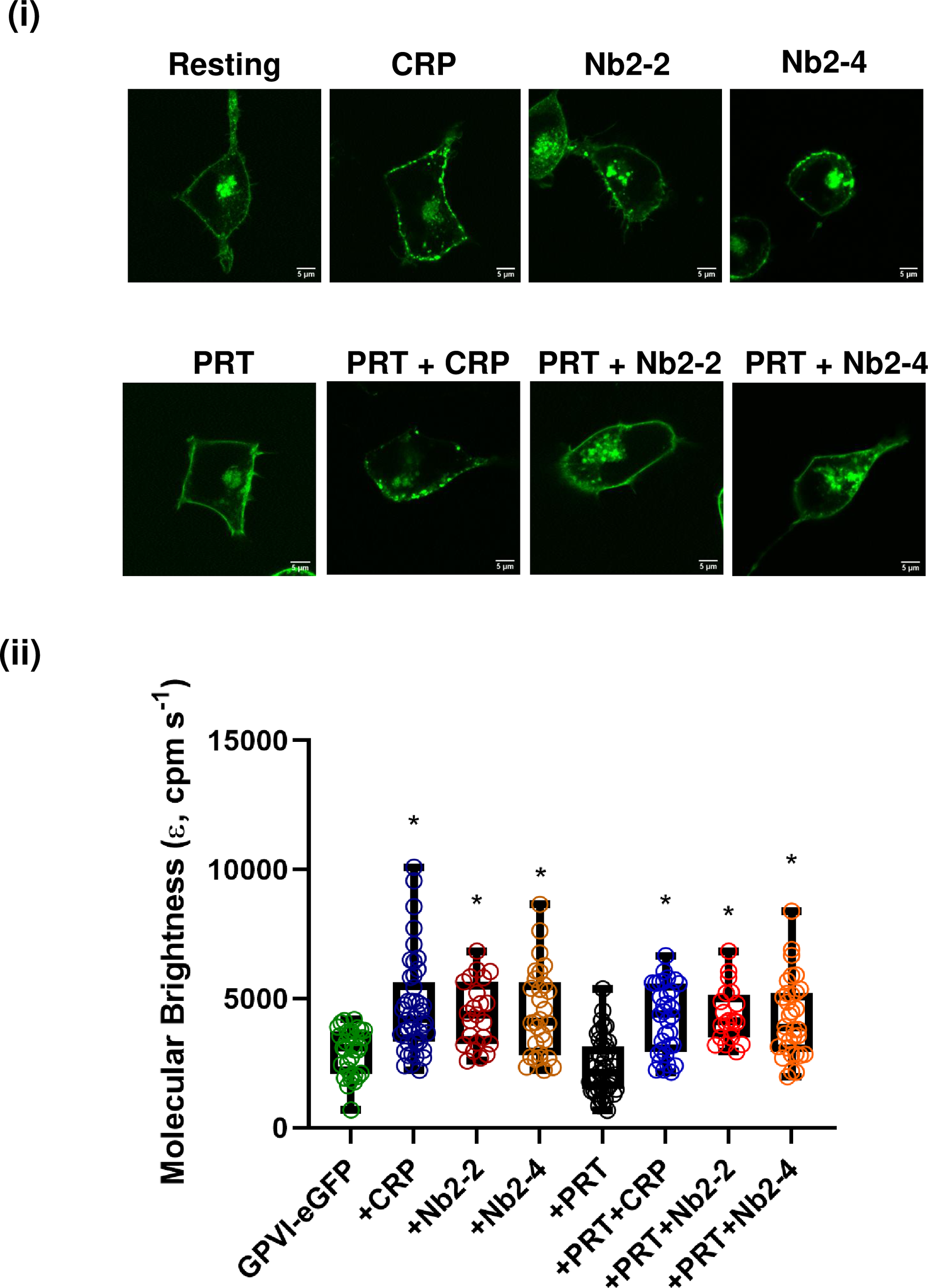

Supplement: S5 Fig — (i) Representative confocal microscopy images showing membrane localisation of GPVI-eGFP + FcRγ-chain resting and treated with collagen-related peptide (CRP, 10 μg/ml), divalent nanobody (Nb) Nb2-2 (10 nM), tetravalent Nb2-4 (10 nM), PRT-060318 (PRT) (10 μM) and PRT + multivalent ligands as labelled, in transfected HEK293T cells (field of view = 52 x 52 μm) (scale bar = 5 μm). (Aii) Box plots showing the effect of CRP (10 μg/ml), divalent Nb2-2 (10 nM), tetravalent Nb2-4 (10 nM), PRT (10 μM) and PRT + multivalent ligands on the molecular brightness (cpm s-1) of GPVI-eGFP. For all box plots, centre lines represent the median; box limits indicate the 25th and 75th percentiles and whiskers extend to minimum and maximum points. Significance was measured with Kruskal-Wallis with Dunn’s post-hoc where P ≤ 0.05. * = significance compared to GPVI alone (no ligand). FCS measurements were taken in 20–47 cells (n = 3). (TIF) [file pcbi.1010708.s006.tif]

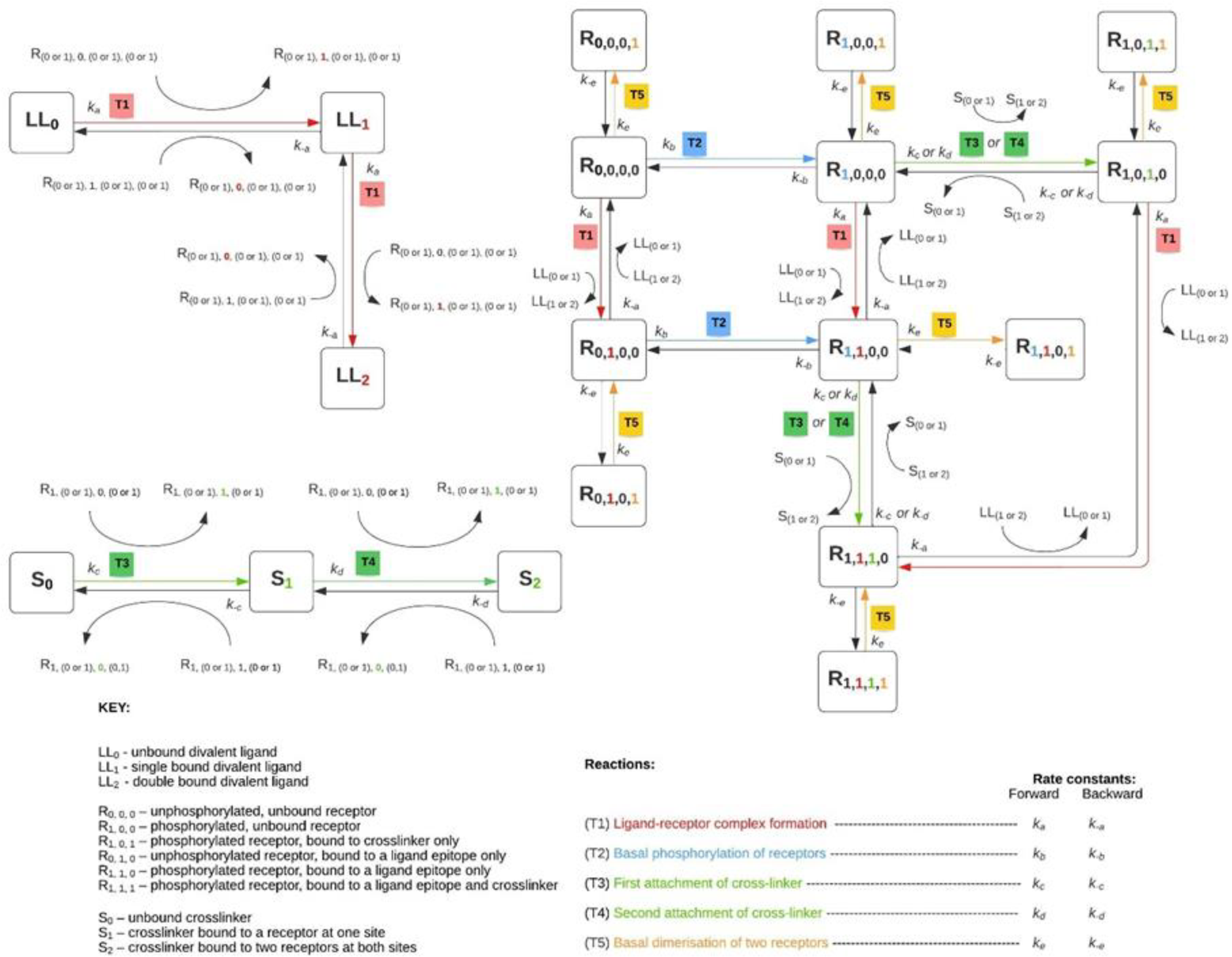

Supplement: S6 Fig — The Figure illustrates all possible states of all species comprising the ABM model and how these change based on inter as well as intra species interactions. There are six reactions occurring on three species: a divalent ligand (L2), receptor (R) and a crosslinker (S). A ligand-receptor complex formation reaction is coloured red, receptor phosphorylation (either basal or post ligand attachment to a receptor) is coloured blue, receptor-crosslinker complex formation is coloured green (either to an unbound or partially bound crosslinker), and receptor-receptor dimerisation reaction is coloured yellow. (TIF) [file pcbi.1010708.s007.tif]

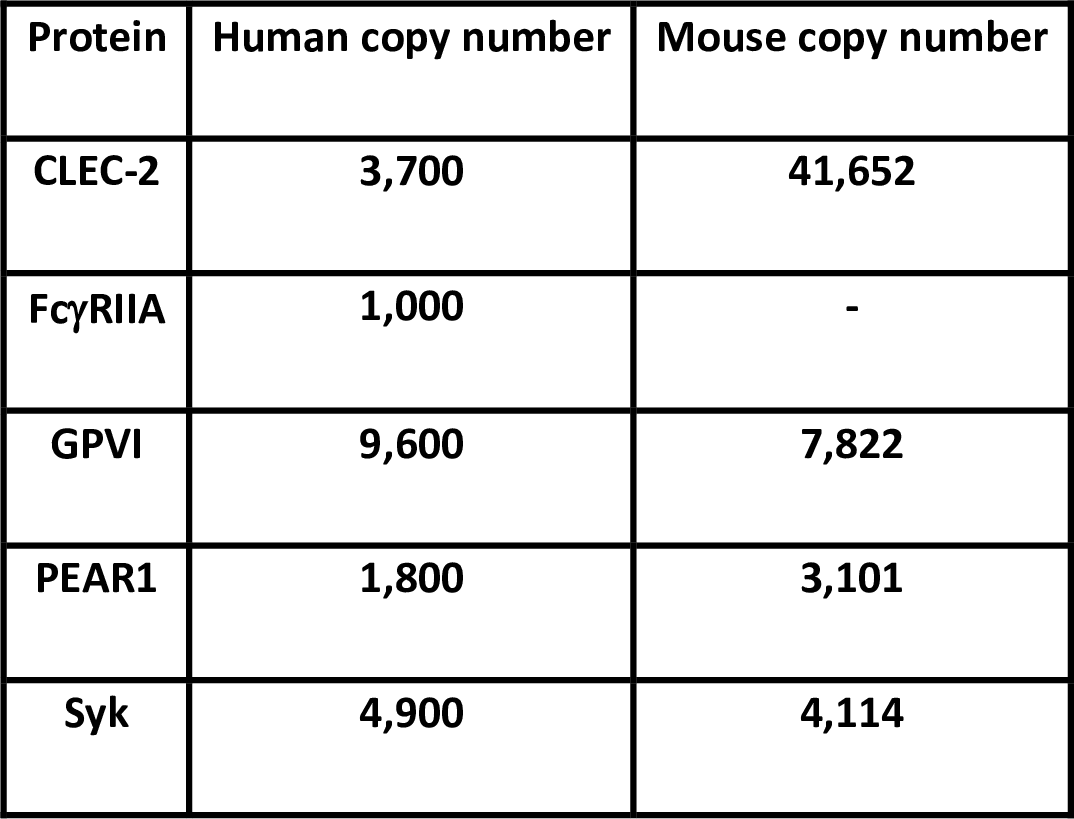

Supplement: S1 Table — The expression levels of monomeric receptors and Syk. The levels are taken from quantitative proteomic studies in human [51] and mouse platelets [52]. (TIF) [file pcbi.1010708.s008.tif]

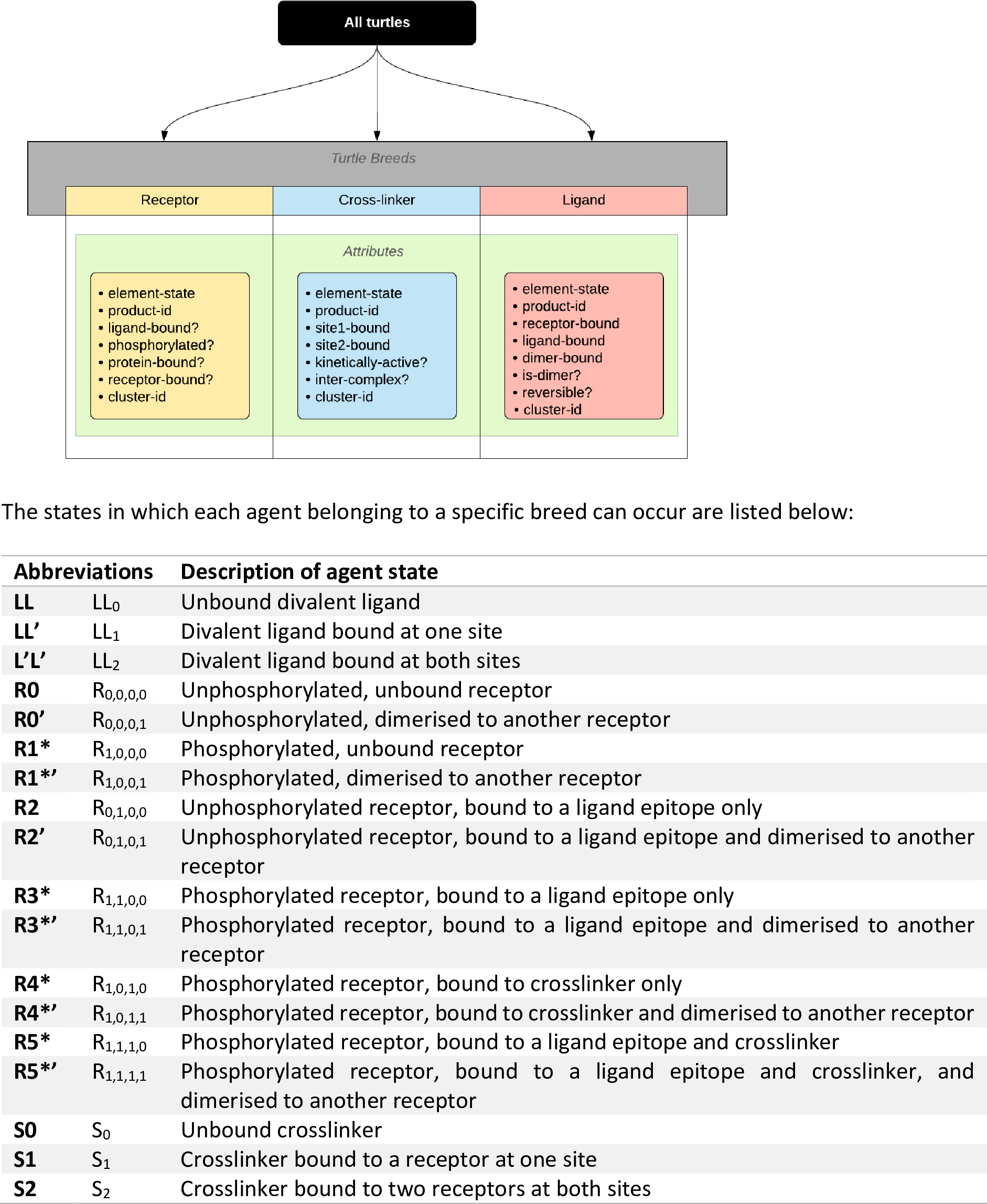

Supplement: S2 Table — All turtles are differentiated into three main breeds namely ligands, receptors and cytosolic cross-linkers. Each breed possesses a unique set of attributes that can be changed. The attributes are responsible for conversion of the state of each agent. The main attributes for each breed are listed in the Table below. (TIF) [file pcbi.1010708.s009.tif]
